# Taxonomy and phylogeny of saprobic Sordariomycetes (Ascomycota) from woody litter in terrestrial ecosystems of Yunnan Province, China

**DOI:** 10.3897/mycokeys.130.181430

**Published:** 2026-03-18

**Authors:** Guang-Cong Ren, Dhanushka Wanasinghe, Heng Gui, Zu-Yi Zhou, Xian Shi, Turki Kh. Faraj, Kai-Xuan Dong

**Affiliations:** 1 School of Pharmacy, Guiyang Kangyang University, Guiyang 550081, China School of Pharmacy, Guiyang Kangyang University Guiyang China; 2 Department of Economic Plants and Biotechnology, Yunnan Key Laboratory for Wild Plant Resources, Kunming Institute of Botany, Chinese Academy of Sciences, Kunming 650201, China Center for Mountain Futures, Kunming Institute of Botany, Chinese Academy of Sciences Honghe China; 3 Center for Mountain Futures, Kunming Institute of Botany, Chinese Academy of Sciences, Honghe 654400, China Yunnan Key Laboratory for Wild Plant Resources, Kunming Institute of Botany, Chinese Academy of Sciences Kunming China; 4 Department of Soil Science, College of Food and Agriculture Sciences, King Saud University, P.O. Box 145111, Riyadh 11362, Saudi Arabia College of Food and Agriculture Sciences, King Saud University Riyadh Saudi Arabia

**Keywords:** 3 new species, 7 new records, Barrmaeliaceae, Diaporthomycetidae, Graphostromataceae, Lasiosphaeridaceae, Linocarpaceae, Neoleptosporellaceae, Sordariomycetidae

## Abstract

As part of our investigation into the diversity of woody litter fungi in Yunnan Province, China, we identified ten microfungal taxa associated with dead woody litter in terrestrial habitats. Based on detailed morphological comparisons and multigene phylogenetic analyses using Maximum Likelihood and Bayesian Inference, including non-translated loci and protein-coding regions, we identified three taxa as new species and seven collections as new records within Sordariomycetes. Three novel species are *Barrmaelia nixiensis*, *Biscogniauxia xishuangbannaensis*, *Neolinocarpon lincangense*, while *Jattaea algeriensis*, *Lasiosphaeris hispida*, *Neoleptosporella camporesiana*, *Planisphaeria karsti*, *P. reniformispora*, *Phaeoacremonium camporesii* and *Thyridium tiliae* represent new host records. Comprehensive morphological descriptions and illustrations are provided to support the identification and classification of these taxa.

## Introduction

Woody litter is vital in forests, playing a key role in nutrient cycling and ecosystem balance (Bucher et al. 2004; Monkai et al. 2013; Marcot 2017). Fungi on the wood such as lignicolous fungi break down these woody litter, releasing essential nutrients into the environment (Boddy 2001; Bucher et al. 2004; Bebber et al. 2006; Lustenhouwer et al. 2020). Fungi also form beneficial partnerships with plants, like mycorrhizal associations, enhancing nutrient absorption and promoting plant growth (Fukasawa et al. 2011; Fukasawa 2021; Delavaux et al. 2023). They produce antimicrobial compounds, aiding in pathogen suppression (Lonsdale et al. 2008; Hyde et al. 2019; Sułkowska-Ziaja et al. 2023). Despite their crucial ecological roles, fungal diversity and interactions with host associations remain poorly understood. Therefore, there is a growing interest in exploring various ecosystems to uncover unidentified fungal species and better understand their ecological roles (Wanasinghe et al. 2024, 2025).

Sordariomycetes is a highly diverse class of fungi within the phylum Ascomycota, playing crucial roles across various ecosystems (Huang et al. 2021). They serve as valuable biocontrol agents in agriculture and contribute significantly to pharmaceutical and biotechnological industries through the production of bioactive secondary metabolites (Hyde et al. 2019, 2024a; Chen et al. 2023). The majority of sordariomycetous species are characterized by perithecial ascomata and inoperculate unitunicate asci, while their asexual morphs are primarily hyphomycetous or coelomycetous (Maharachchikumbura et al. 2016; Samarakoon et al. 2022). This class includes seven subclasses, 56 orders, 215 families, and 1,924 genera (including 390 genera of uncertain placement) (Hyde et al. 2024b), exhibiting higher taxonomic diversity. Sordariomycetes occupy a wide range of ecological niches, live as pathogens, endophytes, saprobes, epiphytes, coprophilous organisms, fungicolous fungi, mycoparasites, lichenized or lichenicolous species (Maharachchikumbura et al. 2015, 2016; Samaradiwakara et al. 2023). They thrive in both aquatic (freshwater and marine) and terrestrial habitats, with terrestrial environments being the most common (Maharachchikumbura et al. 2015; Chen et al. 2023). This global distribution underscores the adaptability and ecological versatility of Sordariomycetes, making them integral components of diverse ecosystems.

Although Sordariomycetes were traditionally classified using morphology (ascomata, asci, ascospores and asexual morph traits) (Gautam et al. 2022), morphology alone is often constrained by plasticity, character overlap, and convergent evolution and asexual morphs are frequently absent or inconsistent (Samaradiwakara et al. 2023; Ekanayaka et al. 2025). Consequently, multi-locus phylogenetic data have become essential for accurate delimitation and evolutionary inference (Luo et al. 2019; Hyde et al. 2020b; Huang et al. 2021; Wang et al. 2024). Phylogenetic investigations based on multilocus DNA sequence data have shed light on the complex relationships among various taxa within the class (Samarakoon et al. 2023; Hyde et al. 2024b), facilitating major taxonomic revisions and the discovery of previously unrecognized lineages (Samarakoon et al. 2022, 2023; Bao et al. 2023; Yang et al. 2023). Given the paucity of distinctive morphological traits in several genera, such molecular-based analyses have become indispensable for accurate taxonomic placement.

Yunnan Province, with its complex topography, diverse climates, and rich vegetation, is a hotspot for fungal diversity (Feng and Yang 2018). Taxonomic studies of ascomycetes in the region have intensified in recent years, yielding over 300 novel species in the past five years alone (Phookamsak et al. 2024). We have conducted extensive field surveys over the past seven years, targeting lignicolous Ascomycota from woody litter (Ren et al. 2022a, 2022b, 2024). This study aims to document previously unaccounted sordariomycetous taxa from our collections, integrating morphological characteristics with multigene phylogenetic analyses. As a result, we introduce three novel species viz. *Barrmaelia nixiensis*, *Biscogniauxia xishuangbannaensis*, *Neolinocarpon lincangense*, and alongside seven new records for the region: *Jattaea algeriensis*, *Lasiosphaeris hispida*, *Neoleptosporella camporesiana*, *Planisphaeria karsti*, *P. reniformispora*, *Phaeoacremonium camporesii* and *Thyridium tiliae*.

## Materials and methods

### Sample collection, observation and isolation

Decayed woody samples were collected from mixed forest areas in China (Yunnan Province), in July 2019 and from July to December 2020, and brought to the laboratory in separate zip-lock plastic bags. Specimens were examined using a stereomicroscope (Olympus SZ61, Tokyo, Japan). Micro-morphological characteristics (i.e. ascomata, peridium, paraphyses, asci and ascospores) were photographed using a Canon EOS 600D (Tokyo, Japan) digital camera mounted on a Nikon ECLIPSE 80i (Tokyo, Japan) compound microscope. All microscopic measurements were taken using the Tarosoft (R) Image Frame Work v.09, and the measurements were reported as minimum–maximum values and average values. Images were processed with Adobe Photoshop CS6 software v.13 (Adobe Systems, San Jose, CA, USA). Single-spore isolation was used to obtain pure cultures, following the methods described by Senanayake et al. (2020). Herbarium materials were deposited at the Cryptogams Kunming Institute of Botany, Academia Sinica (**HKAS**), Kunming, China, and living cultures were deposited at the Kunming Institute of Botany Culture Collection (**KUNCC**), Kunming, China. Faces of fungi (Jayasiri et al. 2015) and Index Fungorum (2026) numbers were obtained for the new taxa.

### DNA extraction, PCR amplification, and sequencing

Genomic DNA was extracted from the mycelium grown on potato dextrose agar (PDA) at 25 °C for four weeks using Biospin Fungus Genomic DNA Extraction Kit (BioFlux®) (Hangzhou, P. R. China). Six gene regions, including internal transcribed spacer region (ITS), large subunit nuclear ribosomal (LSU), small subunit ribosomal RNA (SSU), translation elongation factor 1-alpha gene (*tef*1-α), RNA polymerase II second largest subunit (*rpb*2) and β-tubulin (*tub*2) were amplified with primers ITS5/ ITS4 (White et al. 1990), LR0R/ LR5 (Vilgalys and Hester 1990), NS1/ NS4 (White et al. 1990), 983F/ 2218R (Rehner and Buckley 2005), fRPB2-5F/ fRPB2-7cR (Liu et al. 1999) and T1/ T22 (O’Donnell and Cigelnik 1997), respectively. The PCR thermal cycle programs for SSU, LSU, ITS, *tef*1-α and *rpb*2 were set as described in Wanasinghe et al. (2021). The quality of PCR products was checked on 1% agarose gel electrophoresis stained with ethidium bromide. The PCR products were sent for sequencing at Tsingke Company, Kunming City, Yunnan Province, China. The sequences were deposited in GenBank.

### Phylogenetic analyses

BLASTn searches were performed, and sequences showing high similarity were identified to determine their closest taxonomic matches. The sequences were downloaded from GenBank (http://www.ncbi.nlm.nih.gov/) based on the BLASTn results and recent publications (Huang et al. 2021; Zhang et al. 2021, 2023c, 2025; Sugita and Tanaka 2022; Chen et al. 2024; Crous et al. 2024; Leonardi et al. 2024; Habib et al. 2025; Lim et al. 2025; Luo et al. 2025). The newly generated sequences in this study were assembled by BioEdit 7.2.3 (Hall 1999). The individual gene regions were separately aligned in MAFFT v.7 web server (http://mafft.cbrc.jp/alignment/server/) (Katoh et al. 2019). The alignments of each gene were improved by manually deleting the ambiguous regions plus gaps and combined using BioEdit 7.2.3. The final alignments were converted to NEXUS format (nxs) using Clustal X version 1.81 (Thompson et al. 1997) and processed for Bayesian and maximum parsimony analysis. The FASTA format was changed into PHY format via Alignment Transformation Environment (ALTER) online program (http://www.sing-group.org/ALTER/) and used for maximum likelihood (ML) analysis. The maximum likelihood (ML) analysis was performed on the CIPRES Science Gateway v.3.3 (http://www.phylo.org/portal2/; Miller et al. 2010) using RAxML-HPC2 on XSEDE v.8.2.12 (Stamatakis 2014) with parameters adjusted for 1000 bootstrap iterations and the GTRGAMMA substitution model. Bayesian inference was performed in MrBayes v.3.2.7a (Ronquist et al. 2012) using Markov chain Monte-Carlo sampling (BMCMC) to determine posterior probabilities (PPs) (Rannala and Yang 1996). The model of evolution for each gene was estimated using MrModeltest v.2.3 (Nylander et al. 2008) via PAUP v.4.0b10 (Ronquist and Huelsenbeck 2003). Six simultaneous Markov chains were run for 2,000,000 generations, with trees sampled at every 200 generations, until it was stopped when the standard deviation of split frequencies between the two simultaneous runs dropped below 0.01. Phylogenetic trees were visualized with FigTree v.1.4.0 (Rambaut 2012) and edited using Microsoft PowerPoint and Adobe Illustrator® CS6 v.26.0 (Adobe Systems, San Jose, CA, USA).

## Results

### Taxonomy and phylogeny

Based on polyphasic approaches, a total of ten species were indentified, including three new species and seven new records, updated phylogenetic trees and descriptions for all these taxa are given below. The classification follows Hyde et al. (2024b) and Thiyagaraja et al. (2025).

#### Diaporthomycetidae Senan., Maharachch. & K.D. Hyde, 2015

**Calosphaeriales M.E. Barr, Mycologia 75(1): 11 (1983)**

##### Calosphaeriaceae

Taxon classificationFungiCalosphaerialesCalosphaeriaceae

Munk, Dansk bot. Ark. 17(no. 1): 278 (1957)

23CC44A2-D7A4-592F-81BB-565EAA3D7810

###### Notes.

Calosphaeriaceae was originally established by Munk (1957) and has since undergone several taxonomic revisions (Damm et al. 2008; Réblová et al. 2015; Maharachchikumbura et al. 2016; Wijayawardene et al. 2018). Phylogenetic studies confirmed the inclusion of *Calosphaeria*, *Flabellascus*, *Jattaea*, and *Togniniella* within this family (Huang et al. 2021; Hyde et al. 2024b). Generally, Calosphaeriaceae species are saprobic, inhabiting dead wood and bark of various trees and shrubs. However, some species of *Jattaea* and *Calosphaeria* have also been associated with fruit trees, especially *Prunus* species, where they are implicated in cankers and necrotic lesions on woody tissues (Kazemzadeh-Chakusary et al. 2020). In this study, we introduced a new host record, *Jattaea algeriensis* from *Myrsine seguinii* (Myrsinaceae).

##### Jattaea algeriensis

Taxon classificationFungiCalosphaerialesCalosphaeriaceae

Berl., Icon. fung. (Abellini) 3(1–2): 6 (1900)

EF527B3D-E5C3-5373-9092-D0F2409748D7

Index Fungorum: IF215105

Facesoffungi Number: FoF10117

Fig. 2

###### Description.

***Saprobic*** on dead woody twigs. **Sexual morph: *Ascomata*** perithecia, 150–320 µm high × 135–430 µm diam. (x̄ = 210 × 290 µm, n = 5), scattered, solitary on wood beneath the periderm, dark brown to black, multi-loculate, each locule 120–170 µm high × 140–190 µm diam. (x̄ = 140 × 160 µm, n = 5), with small papilla on the host surface, with cylindrical neck, central or lateral. ***Ostioles*** 100–170 µm high × 40–70 µm diam. (x̄ = 139 × 53 µm, n = 5), ostiolum periphysate. ***Peridium*** 8–15 µm thick, leathery, 3–5 layers of brown cells of ***textura angularis***. ***Paraphyses*** 2–3 µm wide, numerous, hyaline, filiform, unbranched, septate, guttulate, embedded in a gelatinous matrix, slightly tapering towards the apex, obtuse. ***Asci*** 20–32 × 5–6.3 μm (x̄ = 27 × 5.5 μm, n = 20), 8-spored, unitunicate, short-stipitate, clavate, broadly rounded at the thickened apex, tapering towards the base. ***Ascospores*** 4.5–6.4 × 1.7–2.2 μm (x̄ = 5.4 × 2 μm, n = 30), 2–3-seriate, aseptate, ellipsoidal to suballantoid, hyaline, 2-guttulate, smooth-walled. **Asexual morph**: Undetermined.

**Figure 1.**
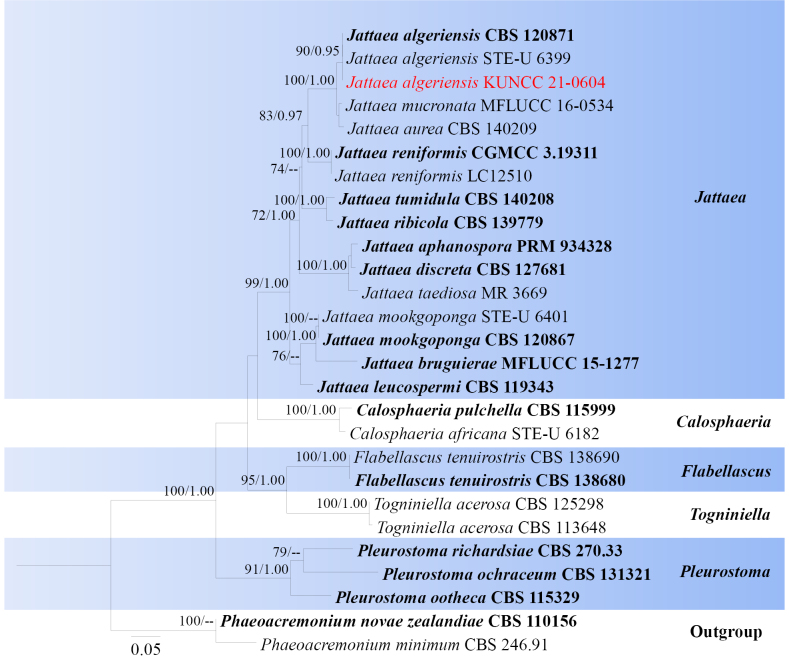
Phylogram generated from ML analysis based on LSU, ITS and TUB sequence data representing Calosphaeriaceae. Related sequences are obtained following Zhang et al. (2021). Twenty-seven strains are included in the combined analyses, which comprise 1834 characters for LSU, ITS and TUB alignment. *Phaeoacremonium novae zealandiae* (CBS 110156) and *P. minimum* (CBS 246.91) were used as the outgroup taxa. The best-scoring RAxML tree with a final likelihood value of -10057.676635 is presented. The matrix had 618 distinct alignment patterns, with 20.59% of undetermined characters or gaps. Estimated base frequencies were as follows; A = 0.222567, C = 0.280141, G = 0.295349, T = 0.201943; substitution rates AC = 1.276096, AG = 2.336664, AT = 1.587462, CG = 0.904375, CT = 4.777312, GT = 1.0000. Gamma distribution shape parameter *α* = 0.182504 and tree-length = 1.960861. The tree topology of the ML analysis is similar to the Bayesian analysis. Bootstrap values for ML equal to or greater than 70% and posterior probability values greater than 0.95 from BYPP analysis labelled on the nodes. Strains of the newly described species are in red, while type strains are in bold.

**Figure 2.**
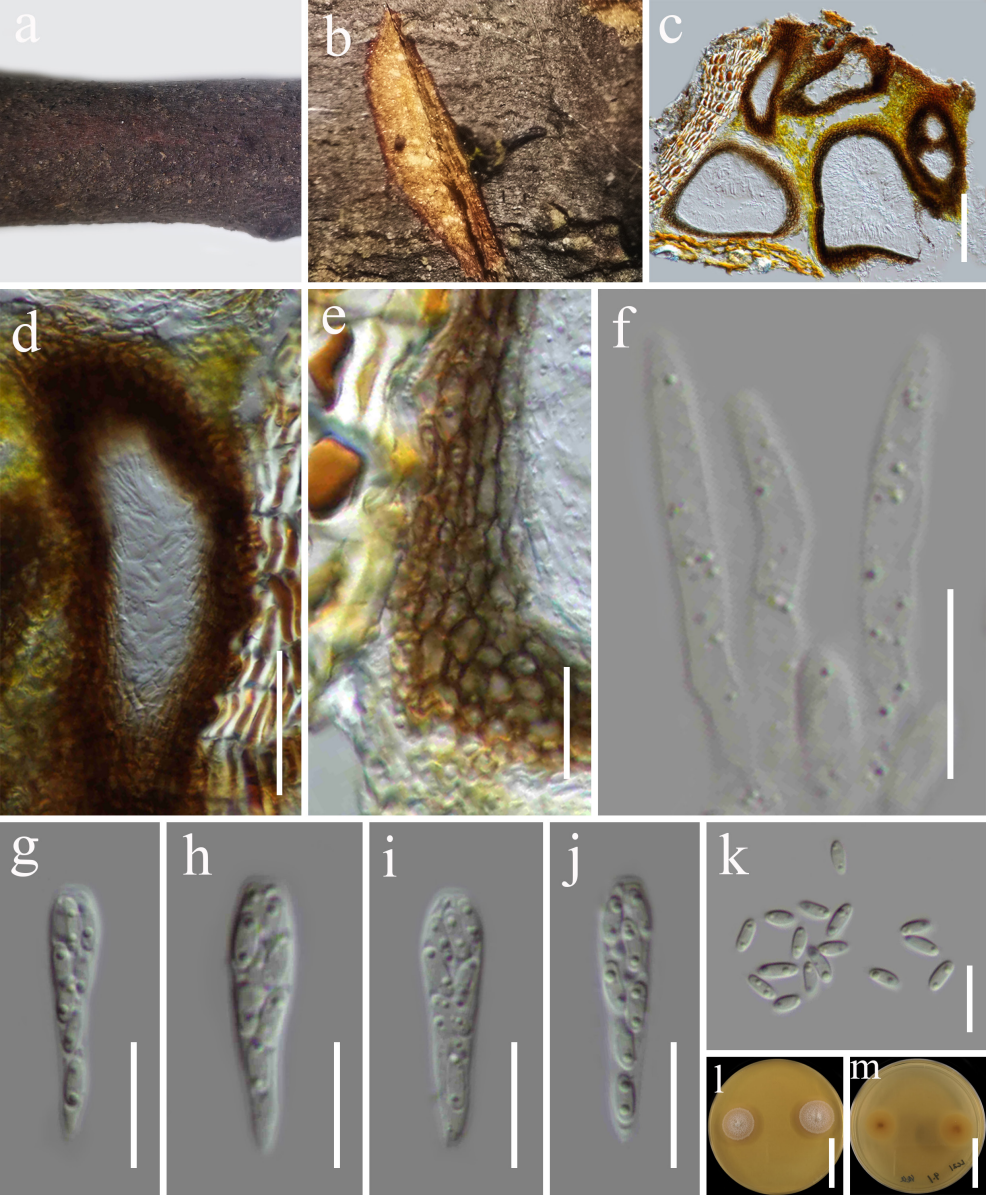
*Jattaea algeriensis* (HKAS 122750). **a, b**. Appearance of ascomata on host substrate; **c**. Section of ascoma; **d**. Section of ostiole; **e**. Peridium; **f**. Paraphyses; **g–j**. Asci; **k**. Ascospores; **l, m**. Culture characters on PDA (**l** from above; **m** from below). Scale bars: 100 μm (**c**); 50 μm (**d**); 10 μm (**e**); 10 μm (**f–k**); 20 mm (**l, m**).

###### Culture characteristics.

Ascospores germinating on PDA within 24 h at room temperature (25 °C). Germ tubes are produced from the basal and apical cell of ascospore. Colonies on PDA, slow growth, reaching 15 mm diameter after one week at 20–25 °C, mycelia superficial, circular, fimbriate, flat, entire edge, white; reverse, yellowish.

###### Material examined.

China • Yunnan Province, Lincang, (24°5'30"N, 100°5'33"E, elevation: 1557.5 m) on dead woody twigs of *Myrsine seguinii* (Myrsinaceae), 10 August 2020, G.C. Ren, LC21 (HKAS 122750), living culture KUNCC 21-0604.

###### Known distribution and hosts.

Algeria, on decayed canes of *Rubus fruticosus* (Saccardo and Sydow 1902; Réblová 2011); South Africa, on wood of *Prunus salicina* (Damm et al. 2008; Réblová 2011); Iran, as pathogens derived from *Mespilus germanica* and *Parrotia persica* (Kazemzadeh-Chakusary et al. 2020); China, on dead woody twigs of *Myrsine seguinii* (Myrsinaceae) (this study).

###### GenBank numbers.

ITS: PX568860, LSU: PX473152, *tef*1-α: PX521731.

###### Notes.

*Jattaea algeriensis* was initially described by Saccardo and Sydow (1902) based on its sexual morphic characteristics. Later, Réblová (2011) synonymized *Jattaea prunicola* with *J. algeriensis* following detailed morphological analysis and phylogenetic studies. *Jattaea prunicola*, originally introduced by Damm et al. (2008), was distinguished by its sexual morph and a phialophora-like anamorph observed in pure culture. Recently, *J. algeriensis* was isolated from the necrotic woody tissues of *Mespilus germanica* and *Parrotia persica* in Iran, identified based on both asexual morph features and phylogenetic analysis (Kazemzadeh-Chakusary et al. 2020). According to the multi-gene phylogenetic analyses of combined LSU, ITS, TUB sequence dataset, our new isolate (KUNCC 21-0604) nested with *Jattaea algeriensis* (CBS 120871, STE-U 6399) with 90% ML and 0.95 BYPP bootstrap support (Fig. 1). Our new isolate (KUNCC 21-0604) shares similarities with *Jattaea algeriensis* (CBS 120871, MC 8938) in having perithecia, scattered, solitary, dark brown to black ascomata with papilla; brown cells of textura angularis peridium; unitunicate, clavate, short-stipitate asci; and aseptate, suballantoid, hyaline ascospores. However, there are differences in ascospore and ascomata morphology, *Jattaea algeriensis* (CBS 120871, MC 8938) has hyaline to pale yellow-brown ascospores with an appendage at the base and uni-loculate ascomata, while our new isolate (KUNCC 21-0604) has hyaline ascospores without appendages and multi-loculate ascomata (Damm et al. 2008; Réblová 2011). Sequence comparison for the ITS and LSU region between our isolates (KUNCC 21-0604) and *J. algeriensis* (CBS 120871, STE-U 6399) showed no significant base pair differences. Therefore, we identified our taxon as a new host record of *J. algeriensis* from *Myrsine seguinii* (Myrsinaceae), and new geographic record in China.

#### Togniniales Senan., Maharachch. & K.D. Hyde, in Maharachchikumbura et al., Fungal Diversity 72: 220 (2015)

##### Togniniaceae

Taxon classificationFungiTogninialesTogniniaceae

Réblová, L. Mostert, W. Gams & Crous, Stud. Mycol. 50(2): 540 (2004)

53AA4AE6-49E3-5325-A2ED-1AC3EAF6A70C

###### Notes.

Réblová et al. (2004) established Togniniaceae based on phylogenetic analysis of LSU and SSU sequence. The family include both sexual (*Conidiotheca* and *Togninia*) and asexual genera (*Phaeoacremonium*) (Réblová and Mostert 2007). This family has been included in Calosphaeriales (Mostert et al. 2003) and Diaporthales (Mostert et al. 2006) based on morpho-molecular analysis. Maharachchikumbura et al. (2015) excluded it from Diaporthales and introduced it in Togniniales. Subsequent studies, including those by Hyde et al. (2020a) and Calabon et al. (2021), accepted both *Conidiotheca* and *Phaeoacremonium* within Togniniaceae. In contrast, more recent classifications by Wijayawardene et al. (2022) and Hyde et al. (2024b) recognize only *Phaeoacremonium* as a valid genus within the family.

##### Phaeoacremonium camporesii

Taxon classificationFungiTogninialesTogniniaceae

Wijes., Camporesi & K.D. Hyde, Fungal Diversity 114: 360 (2022)

D7563FCB-6F66-5B02-8D3D-763FFD6F0E2E

Index Fungorum: IF559245

Facesoffungi Number: FoF10568

Fig. 4

###### Description.

***Saprobic*** on dead woody twigs. **Sexual morph: *Ascomata*** 160–220 μm high, 180–360 μm diam. (x̄ = 195 × 270 μm, n = 5), perithecial, immersed to semi-immersed, globose to subglobose, sometimes, truncate at the base, black, coriaceous, ostiolate. ***Peridium*** 10–36 µm thick, multi-layers of brown to dark brown cells of ***textura angularis***. ***Paraphyses*** comprising numerous, 2.5–4.5 μm wide, hyaline, branched, septate, slightly constricted at septa and gradually narrowed towards the apex. ***Asci*** 15–20 × 4–5 μm (x̄ = 17 × 4.4 μm, n = 20), 8-spored, unitunicate, clavate, apex truncate, rounded at the base, apedicellate. ***Ascospores*** 4.4–5.3 × 1–1.4 μm (x̄ = 4.6 × 1.2 μm, n = 30), biseriate, allantoid, reniform with rounded ends, unicellular, hyaline, thin-walled, smooth-walled, often containing small guttules at both ends. **Asexual morph**: Undetermined.

###### Culture characteristics.

Ascospores germinating on PDA within 24 h at room temperature (25 °C). Colonies on PDA, reaching 50–55 mm diameter after one week at 20–25 °C, mycelia superficial, flat, velvety, white, umbonate at center; reverse, white, pale yellow at the center.

###### Material examined.

China • Yunnan Province, Lincang (24°5'30"N, 100°5'33"E, elevation: 1558 m) on dead woody twigs of *Calophyllum polyanthum* (Calophyllaceae), 12 July 2020, G.C. Ren, LC31 (HKAS 122752), living culture KUNCC 21-0608.

###### Known distribution and hosts.

Italy, on dead aerial branch of *Corylus avellana* (Phukhamsakda et al. 2022); Iran, on necrotic wood tissues of *Tamarix ramosissima* (Rahimi‐Nia et al. 2025); China, on dead branch of *Calophyllum polyanthum* (Calophyllaceae) (this study).

###### GenBank numbers.

ITS: PP663079, LSU: PP663086, SSU: PX583841.

###### Notes.

**Figure 3.**
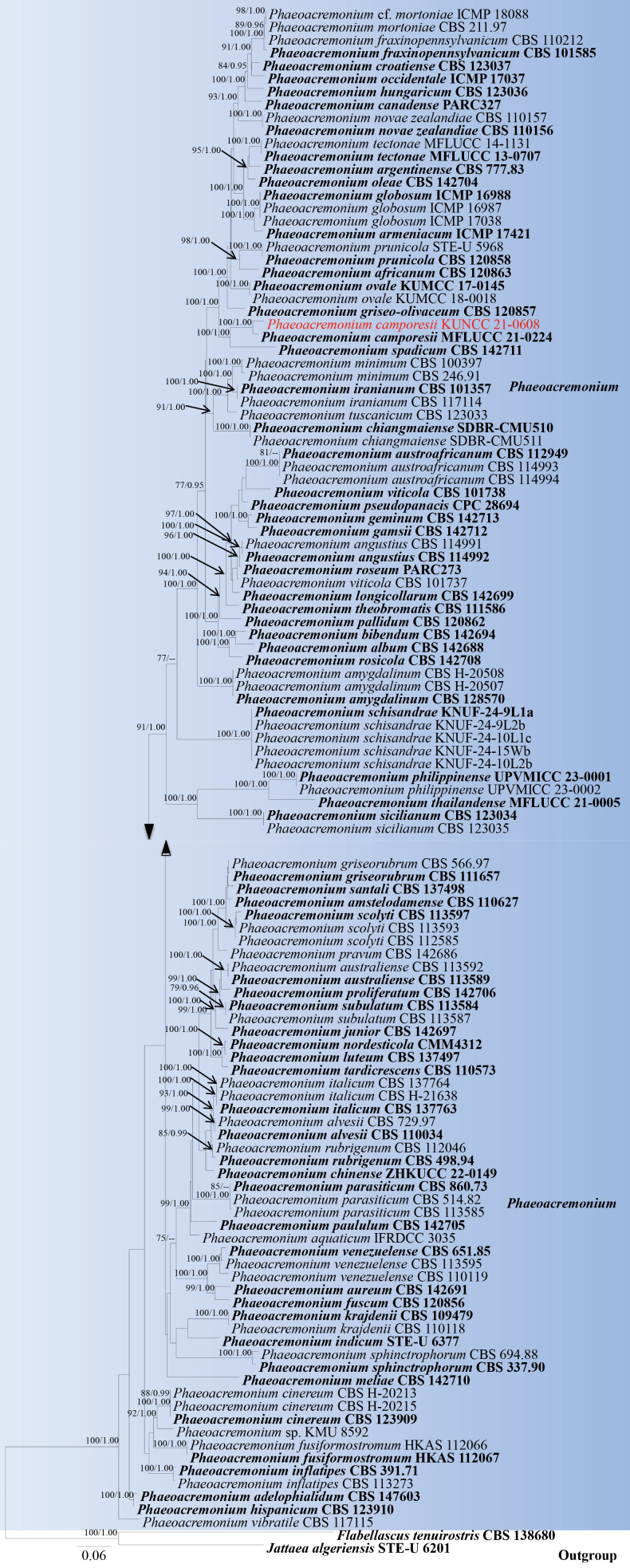
Phylogram generated from ML analysis based on ITS, TUB, ACT, *tef*1-α and LSU sequence data representing *Phaeoacremonium*. Related sequences are obtained following Lim et al. (2025) and Zhang et al. (2025). One hundred and eighteen strains are included in the combined analyses, which comprise 2630 characters for ITS, TUB, ACT, *tef*1-α and LSU alignment. *Flabellascus tenuirostris* (CBS 138680) and *Jattaea algeriensis* (STE-U 6201) were used as the outgroup taxa. The best-scoring RAxML tree with a final likelihood value of -29974.772558 is presented. The matrix had 1369 distinct alignment patterns, with 47.07% of undetermined characters or gaps. Estimated base frequencies were as follows; A = 0.222379, C = 0.293334, G = 0.251529, T = 0.232759; substitution rates AC = 1.395429, AG = 3.734177, AT = 1.396244, CG = 1.142111, CT = 4.959406, GT = 1.0000. Gamma distribution shape parameter α = 0.286131 and tree-length = 4.360818. The tree topology of the ML analysis is similar to the Bayesian analysis. Bootstrap values for ML equal to or greater than 70% and posterior probability values greater than 0.95 from BYPP analysis labelled on the nodes. Strains of the newly described species are in red, while type strains are in bold.

*Phaeoacremonium* was established by Crous et al. (1996), with *P. parasiticum* designated as the type species. Currently, 73 species are recognized within the genus (Calabon et al. 2024; Mostert et al. 2024; Lim et al. 2025; Zhang et al. 2025). Multi-locus phylogenetic analyses based on a concatenated dataset of ITS, TUB, ACT, *tef*1-α, and LSU sequences revealed that our new collection (KUNCC 21-0608) is closely related to *Phaeoacremonium camporesii* (MFLUCC 21-0224), with strong statistical support (100% ML/ 1.00 BYPP; Fig. 3). Comparative analysis of the ITS and TUB sequences between our isolate (KUNCC 21-0608) and the ex-type strain of *P. camporesii* (MFLUCC 21-0224) revealed a 5.4% difference in the TUB region (17/ 313 bp, without gaps), while no significant differences were observed in the ITS region. Morphologically, our new isolate (KUNCC 21-0608) shares similarities with *P. camporesii* in having perithecia, immersed to semi-immersed, globose to subglobose ascomata; unitunicate, 8-spored, clavate asci with a truncate apex; and biseriate, allantoid to reniform, unicellular, hyaline, thin- and smooth-walled ascospores with rounded ends. Based on both molecular and morphological evidence, we identify KUNCC 21-0608 as a new host record of *Phaeoacremonium camporesii*, collected from *Calophyllum polyanthum* (Calophyllaceae) in China.

**Figure 4.**
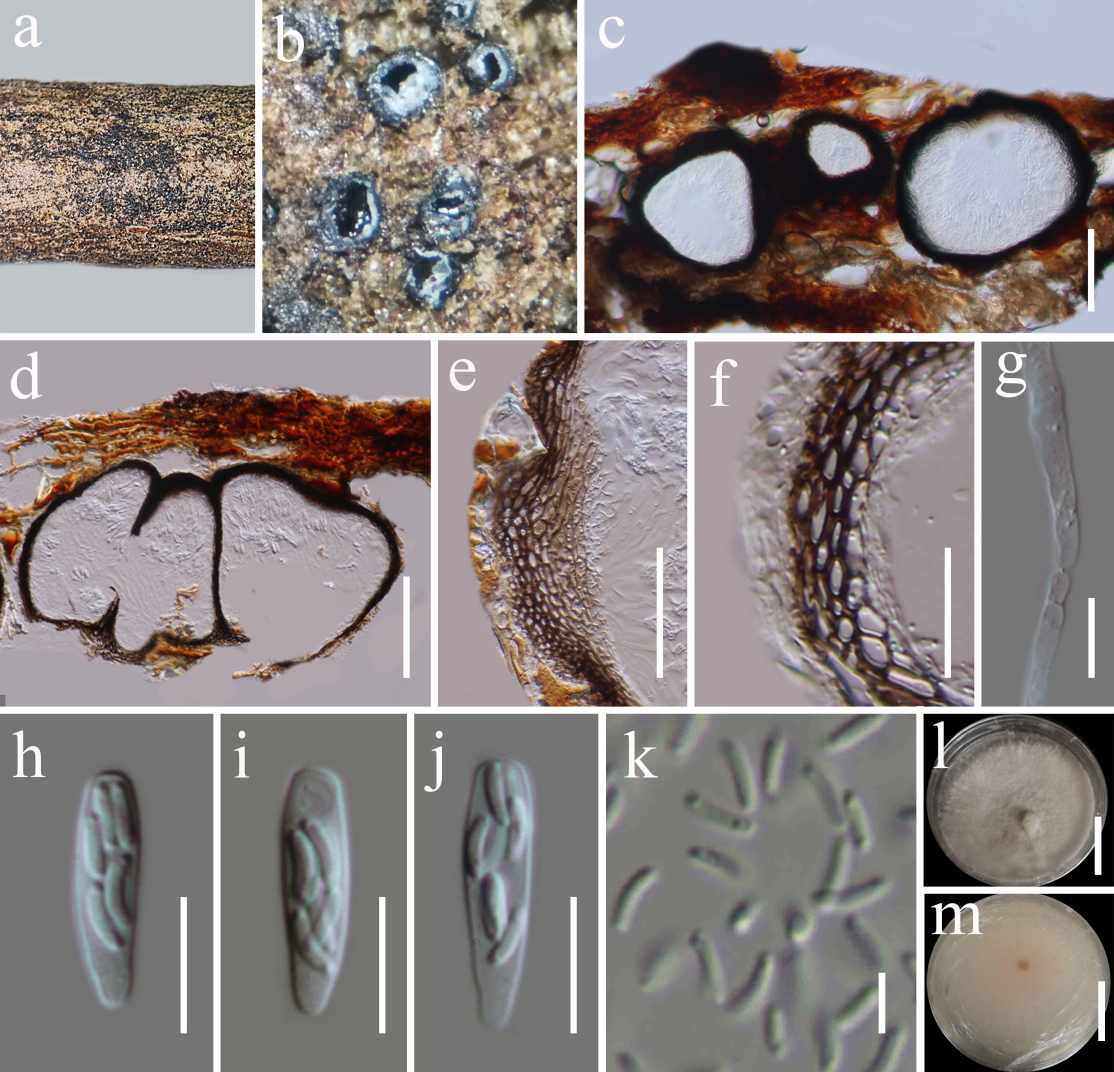
*Phaeoacremonium camporesii* (HKAS 122752). **a**. Appearance of ascomata on host substrate; **b**. Ascomata in transverse section; **c, d**. Sections of ascomata; **e, f**. Peridium; **g**. Paraphyses; **h–j**. Asci; **k**. Ascospores; **l, m**. Culture characters on PDA (**l** from above, **m** from below). Scale bars: 100 μm (**c–e**); 10 μm (**f–j**); 5 μm (**k**); 20 mm (**l, m**).

#### Thyridiales R. Sugita & Kaz. Tanaka, MycoKeys 86: 156 (2022)

##### Thyridiaceae

Taxon classificationFungiThyridialesThyridiaceae

J.Z. Yue & O.E. Erikss., Syst. Ascom. 6(2): 233 (1987)

530383C0-AE05-53D7-91D8-E436C3366B12

###### Notes.

Thyridiaceae was established by Yue and Eriksson (1987) with *Thyridium vestitum* designated as the type species. For a long time, the family was treated as Sordariomycetes*incertae sedis* until Sugita and Tanaka (2022) established a new order, Thyridiales, within Sordariomycetes to accommodate Thyridiaceae. Members of Thyridiaceae are saprobic or hemibiotrophic on woody substrates, with some species occurring as endophytes notably, there are no reports of pathogenic species in this family (Hyde et al. 2020a). At present, Thyridiaceae comprises two genera, *Thyridium* and *Phialemoniopsis* (Hyde et al. 2020b, 2024b).

##### Thyridium tiliae

Taxon classificationFungiThyridialesThyridiaceae

Crous & Akulov, Fungal Syst. Evol. 14: 427 (2024)

70CB494B-A634-5FC5-BCFC-03330F995B88

Index Fungorum: IF856106

Facesoffungi Number: FoF18852

Fig. 6

###### Description.

***Saprobic*** on dead woody twigs. **Sexual morph: *Stromata*** scattered to grouped, subepidermal, immersed to erumpent, with interwoven yellowish or black tomentum. ***Ascomata*** 290–520 μm high, 300–530 μm diam. (x̄ = 410 × 430 μm, n = 5), mostly grouped, immersed in stromata to erumpent through host surface. ***Peridium*** 20–30 µm thick, composed of several layers of thick-walled, dark brown cell of ***textura angularis***. ***Ostiolar*** neck cylindrical, short or long, separated or convergent in upper stromata. ***Paraphyses*** 3–5.4 µm wide, numerous, septate, unbranched, hyaline, cylindrical. ***Asci*** 130–160 × 15–21 μm (x̄ = 148.3 × 18.3 μm, n = 20), 8-spored, unitunicate, cylindrical, rounded at the apex, with a short pedicel. ***Ascospores*** 20–23 × 8–11.3 μm (x̄ = 21 × 10 μm, n = 30), muriform, ellipsoid, hyaline when immature, becoming dark brown at maturity, with 3 transverse septa and 1 longitudinal septum, thickened, and pigmented at the septa. **Asexual morph**: Undetermined.

**Figure 5.**
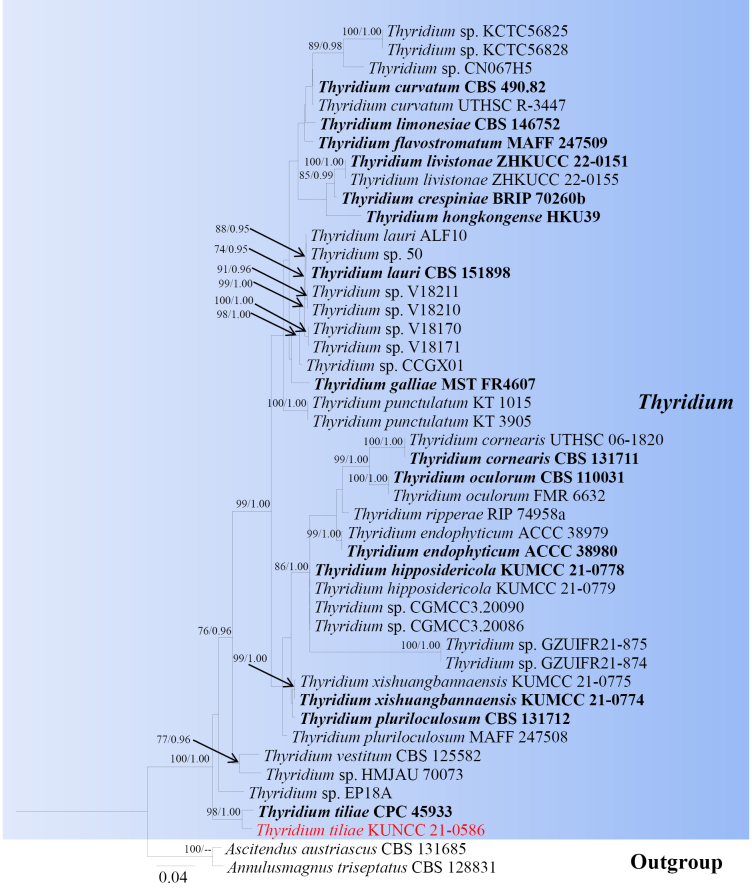
Phylogram generated from ML analysis based on LSU, ITS, ACT, *tef*1-α and TUB sequence data representing *Thyridium*. Related sequences are obtained following Sugita and Tanaka (2022), Leonardi et al. (2024) and Crous et al. (2024). Forty-six strains are included in the combined analyses, which comprise 2630 characters for LSU, ITS, ACT, *tef*1-α and TUB alignment. *Ascitendus austriascus* (CBS 131685) and *Annulusmagnus triseptatus* (CBS 128831) were used as the outgroup taxa. The best-scoring RAxML tree with a final likelihood value of -11734.391258 is presented. The matrix had 812 distinct alignment patterns, with 48.23% of undetermined characters or gaps. Estimated base frequencies were as follows; A = 0.226472, C = 0.271383, G = 0.262638, T = 0.239508; substitution rates AC = 1.067369, AG = 2.011680, AT = 1.164926, CG = 1.259845, CT = 6.000431, GT = 1.0000. Gamma distribution shape parameter α = 0.171126 and tree-length = 0.987258. The tree topology of the ML analysis is similar to the Bayesian analysis. Bootstrap values for ML equal to or greater than 70% and posterior probability values greater than 0.95 from BYPP analysis labelled on the nodes. Strains of the newly described species are in red, while type strains are in bold.

**Figure 6.**
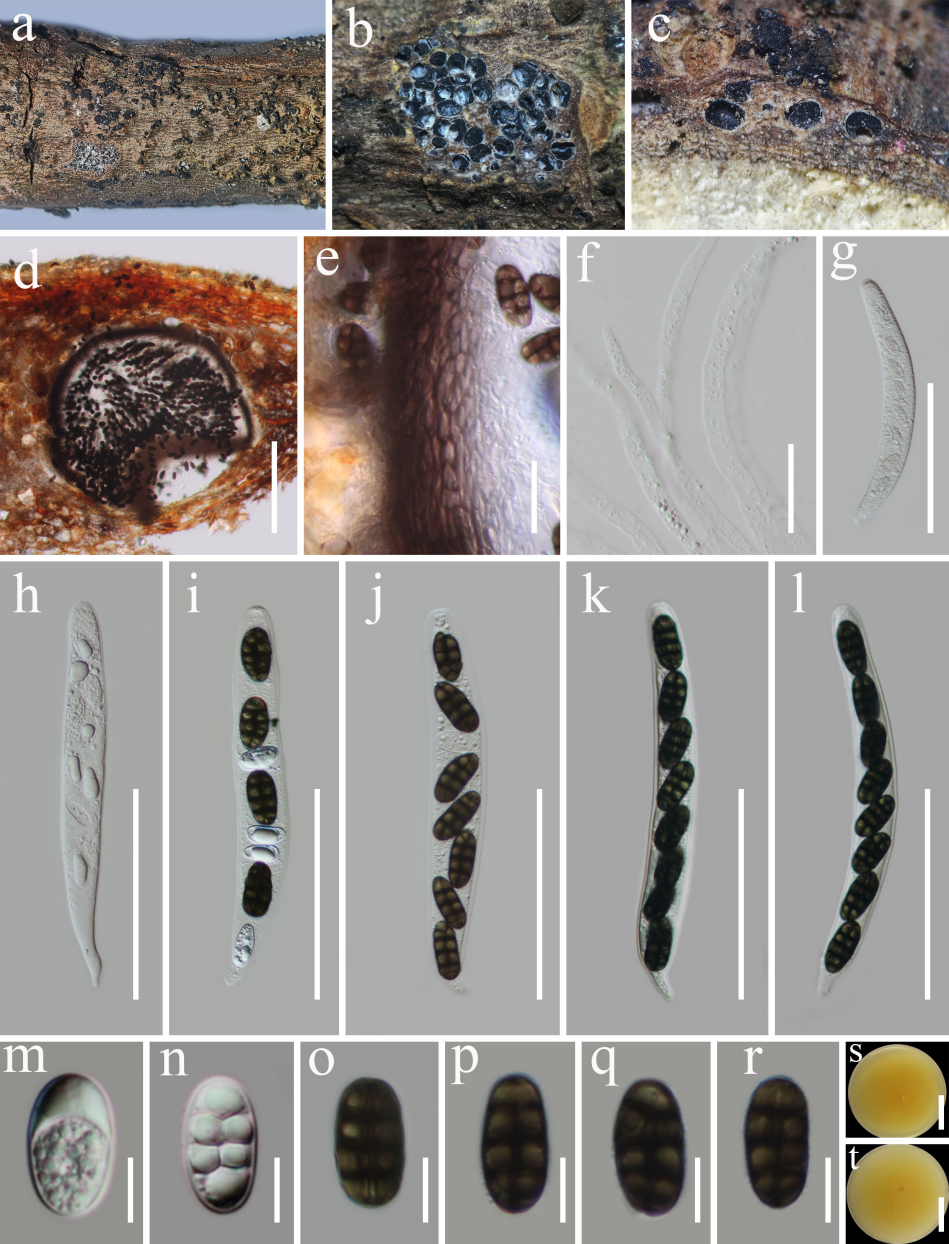
*Thyridium tiliae* (HKAS 122742). **a**. Appearance of ascomata on the host substrate; **b, c**. Ascomata in transverse section; **d**. Section of an ascoma; **e**. Peridium; **f**. Paraphyses; **g**–**l**. Asci; **m**–**r**. Ascospores; **s, t**. Culture characters on PDA (**s** from above; **t** from below). Scale bars: 200 μm (**d**); 200 μm (**e, f**); 100 μm (**g**–**l**); 10 μm (**m**–**r**); 20 mm (**s, t**).

###### Culture characteristics.

Ascospores germinating on PDA within 24 h at room temperature (25 °C). Colonies on PDA, reaching 50–55 mm diameter after one week at 20–25 °C, mycelia superficial, flat, fimbriate, pale yellow, some hyphae are embedded in the culture medium; reverse, pale yellow.

###### Material examined.

China • Yunnan Province, Xishuangbanna Dai Autonomous Prefecture, Jinghong (21°55.19'N, 101°15.24'E, elevation: 522 m), on dead woody twigs of *Pellacalyx yunnanensis* (Rhizophoraceae), 4 March 2020, G. C. Ren, JH43 (HKAS 122742), living culture KUNCC 21-0586.

###### Known distribution and hosts.

Ukraine, on dead twigs of *Tilia* sp. (Tiliaceae) (Crous et al. 2024); China, on dead woody twigs of *Pellacalyx yunnanensis* (Rhizophoraceae) (This study).

###### GenBank numbers.

ITS: PP663078, LSU: PP663085, SSU: PX583842, *tef*1-α: PX521732.

###### Notes.

The genus *Thyridium* was introduced by Nitschke (1867), with *T. vestitum* as the type species (Esfandiari and Petrak 1950). Currently, 51 species are listed in Species Fungorum (31 January 2026), occurring on various plants as saprobic or hemibiotrophic fungi (Crous et al. 2024), DNA sequences are only available for 18 species. Multi-loci phylogenetic analyses based on a concatenated LSU, ITS, ACT, *tef*1-α and TUB sequence dataset show that our new collections (KUNCC 21-0586) formed a close relationship with *Thyridium tiliae* (CPC 45933) as the basal lineage within the genus (98% ML/ 1.00 BYPP; Fig. 5). Comparative analysis of the ITS, LSU and *tef*1-α sequence data between our isolate KUNCC 21-0586 and *T. tiliae* (CPC 45933, ex-type) revealed nucleotide differences of 0.9% (5/ 546 bp, without gaps), 0.6% (5/ 811 bp, without gaps), and 3.9% (34/ 883 bp, without gaps), respectively. Morphologically, our collection *T. tiliae* shares several morphological features, including scattered stromata, perithecial ascomata immersed in stromata, cylindrical and septate paraphyses, unitunicate cylindrical asci with 8 spores, and septate ascospores (Crous et al. 2024). Based on both molecular and morphological evidence, we identify KUNCC 21-0586 as a new host record of *Thyridium tiliae*, collected from *Pellacalyx yunnanensis* (Rhizophoraceae) in China.

#### Sordariomycetidae O.E. Erikss. & Winka, 1997

**Chaetosphaeriales Huhndorf, A.N. Mill. & F.A. Fernández, 2004**

##### Linocarpaceae

Taxon classificationFungiThyridialesLinocarpaceae

S. Konta & K.D. Hyde, Mycosphere 8(10): 1962 (2017)

A4801154-0CFE-534D-B5F3-159DC9F293D0

###### Notes.

Linocarpaceae was established by Konta et al. (2017) to accommodate two genera, *Linocarpon* (the type genus) and *Neolinocarpon*, based on morphological characteristics and multigene phylogenetic analyses using ITS and LSU sequences. This was placed in Chaetosphaeriales based on phylogenetic evidence. Later, Xu et al. (2020) introduced a third genus, *Claviformispora*, into Linocarpaceae, supported by both morphological features and phylogenetic analyses of LSU, SSU and *tef*1-α. *Claviformispora* currently comprises one species, *Linocarpon* 45 species and *Neolinocarpon* 14 species (Luo et al. 2025; Index Fungorum 2026). Species of *Linocarpon* and *Neolinocarpon* are characterized by narrow and long filiform, hyaline ascospores, often with refringent bands. In contrast, *Claviformispora* is distinguished by its clavate ascospores and a bambusicolous habitat (Xu et al. 2020). Linocarpaceae species are associated with plants as saprobes or endophytes and widely distributed (Zhang et al. 2023a).

##### Neolinocarpon lincangense

Taxon classificationFungiThyridialesLinocarpaceae

G.C. Ren & Wanas. sp. nov.

D61D497A-7E30-5D48-A53B-CD6C433F49D6

Index Fungorum: IF904525

Facesoffungi Number: FoF18853

Fig. 8

###### Etymology.

The epithet reflects the location where the holotype was collected.

**Figure 7.**
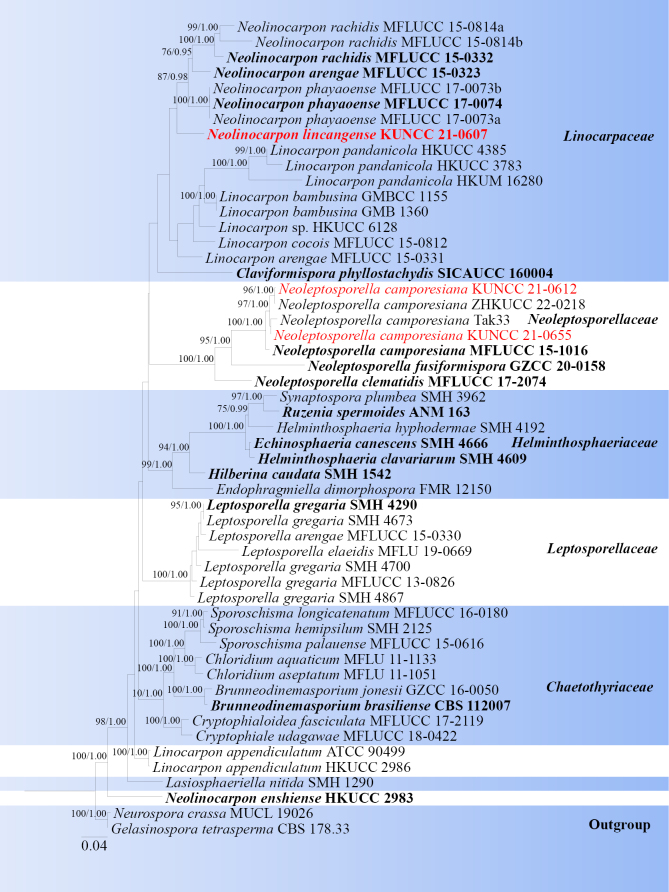
Phylogram generated from ML analysis based on LSU, ITS, ACT, *tef*1-α and TUB sequence data representing Chaetothyriaceae, Helminthosphaeriaceae, Leptosporellaceae, Linocarpaceae and Neoleptosporellaceae. Related sequences are obtained following Zhang et al. (2023c), Luo et al. (2025) and Habib et al. (2025). Forty-six strains are included in the combined analyses, which comprise 2630 characters for SSU, LSU, ITS, and *tef*1-α alignment. *Gelasinospora tetrasperma* (CBS 178.33) and *Neurospora crassa* (MUCL 19026) were used as the outgroup taxa. The best-scoring RAxML tree with a final likelihood value of -20236.771109 is presented. The matrix had 812 distinct alignment patterns, with 48.23% of undetermined characters or gaps. Estimated base frequencies were as follows; A = 0.232490, C = 0.268062, G = 0.305807, T = 0.193641; substitution rates AC = 1.013194, AG = 1.913555, AT = 0.940548, CG = 1.191676, CT = 5.116146, GT = 1.0000. Gamma distribution shape parameter α = 0.317721 and tree-length = 2.260989. The tree topology of the ML analysis is similar to the Bayesian analysis. Bootstrap values for ML equal to or greater than 70% and posterior probability values greater than 0.95 from BYPP analysis labelled on the nodes. Strains of the newly described species are in red, while type strains are in bold.

**Figure 8.**
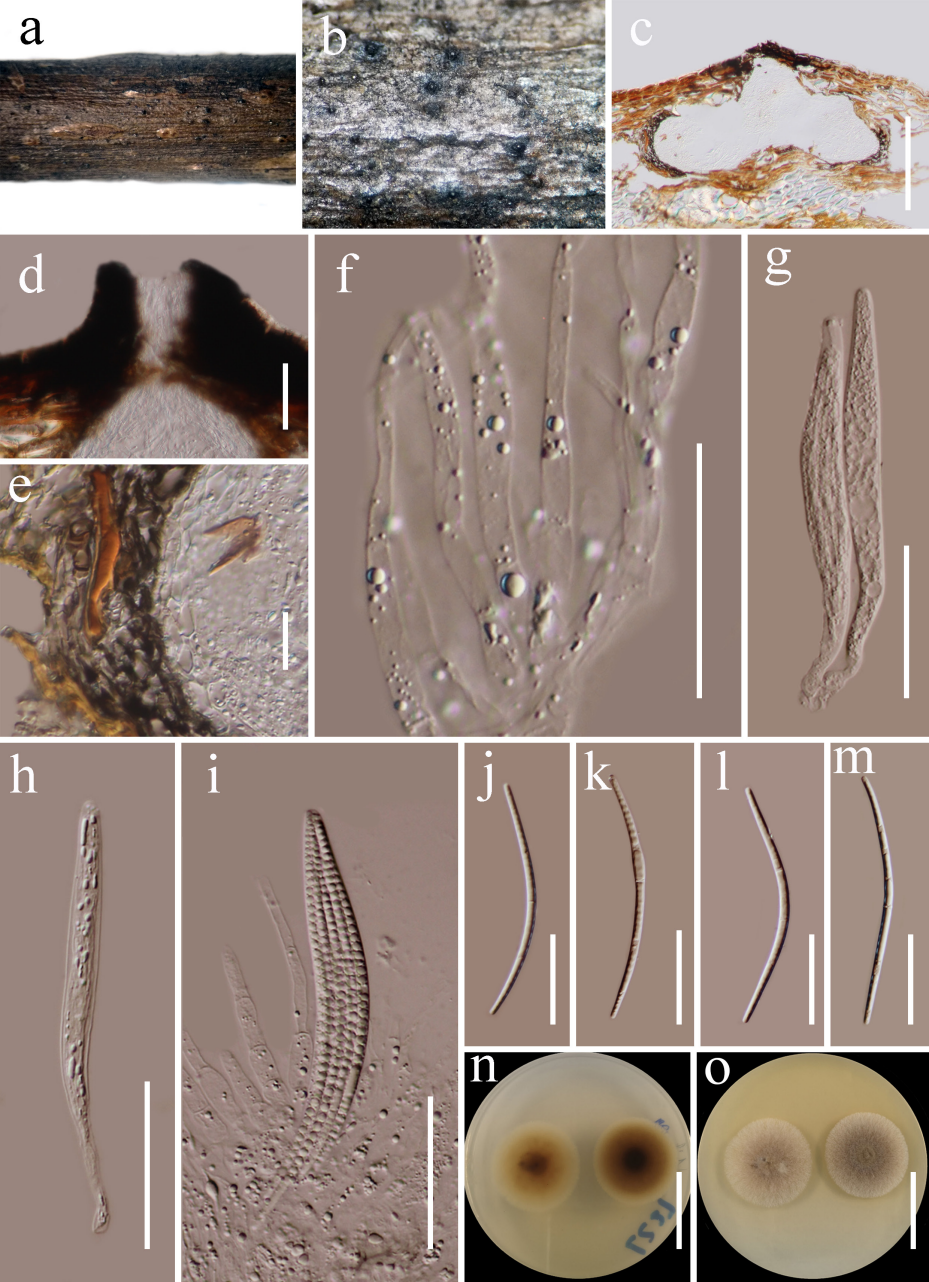
*Neolinocarpon lincangense* (HKAS 122751, holotype). **a, b**. Appearance of ascomata on host substrate; **c**. Section of ascoma; **d**. Section of ostiole; **e**. Peridium; **f**. Hamathecium; **g–i**. Asci; **j–m**. Ascospores; **n, o**. Culture characters on PDA (**o** from above; **n** from below). Scale bars: 200 μm (**c, e**); 50 μm (**d**); 30 μm (**f, g–i**); 40 μm (**j–m**); 30 mm (**n, o**).

###### Holotype.

HKAS 122751.

###### Description.

***Saprobic*** on dead woody twigs. **Sexual morph: *Ascomata*** 190–440 µm high, 400–540 μm diam. (x̄= 330 × 480 μm, n = 5), solitary to gregarious, immersed to semi-immersed, becoming raised to erumpent through the host tissue, globose to subglobose, carbonaceous, black, papillate. ***Ostiolar canal*** 60–120 μm wide, 100–260 μm long, central, cylindrical, straight, periphysate. ***Peridium*** 10–18 μm wide, outer cells fused with the host epidermal cells, thickness on both sides, composed of dark brown outer layer and hyaline inner layer comprising, thick-walled cells of ***textura angularis***. ***Hamathecium*** 3.5–6.5 μm wide, comprising dense, hyaline, septate, unbranched, filamentous, tapering towards the apex, slightly constricted at the septum, verruculose or guttule. ***Asci*** 100–120 × 8–13.5 μm (x̄ = 110 × 10.7 μm, n = 20), 8-spored, unitunicate, with a wedge-shaped, J- subapical ring, cylindrical, long pedicellate, thin-walled. ***Ascospores*** 82–92 × 2–3 μm (x̄ = 88.7 × 2.5 μm, n = 30), parallel in ascus, fasciculate, filiform, hyaline, aseptate, mostly curved, containing refringent septum-like bands, tapering towards the base and with a narrow-rounded apex, smooth-walled. **Asexual morph**: Undetermined.

###### Culture characteristics.

Ascospores germinating on PDA within 24 h at room temperature. Colonies on PDA, reaching 15–20 mm diameter after one week at 20–25 °C, mycelia superficial, circular, flat, fimbriate, entire edge, white at the margin, gray white at the center; reverse, yellowish at the margin, dark brown at the center.

###### Material examined.

China • Yunnan Province, Lincang (24°5'30"N, 100°5'33"E, elevation: 1557.49 m) on dead woody twigs of *Knema furfuracea* (Myristicaceae), 12 July 2020, G.C. Ren, LC27 (holotype, HKAS 122751), ex-type culture KUNCC 21-0607.

###### GenBank numbers.

LSU: PP663084.

###### Notes.

The genus *Neolinocarpon* was introduced to accommodate linocarpon-like species by Hyde (1992), with *N. globosicarpum* as the type species. Recently, Habib et al. (2025) describe a new species, *Neolinocarpon huaxiense*, found on dead bamboo culms. Currently, 15 species are listed in Index Fungorum (2026). Except for *Neolinocarpon arengae*, *N. enshiense*, *N. phayaoense* and *N. rachidis*, sequence data are available in GenBank. Multi-loci phylogenetic analyses based on a concatenated SSU, LSU, ITS, and *tef*1-α sequence dataset show that our strain (KUNCC21-0607) is clustered with *Neolinocarpon* species (Fig. 1). The morphological characteristics of *Neolinocarpon lincangense* is similar to other species by its immersed ascomata with an ostiole, cylindrical asci with a wedge-shaped, J-, and subapical ring and filiform ascospores (Senwanna et al. 2018). However, *Neolinocarpon arengae*, *N. australiense*, *N. calami*, *N. enshiense*, *N. eutypoides*, *N. globosicarpum*, *N. rachidis* differs from our collection by the mucilaginous appendage (Hyde 1992; Hyde et al. 1998; Konta et al. 2017; Senwanna et al. 2018). The other six *Neolinocarpon* species differ from our new collection: *N. attaleae* has filiform -fusoid to clavate ascospores, *N. inconspicuum* has inconspicuous ascomata, *N. nonappendiculatus* has comparatively larger ascospores (114–138 × 2–2.5 μm), *N. nypicola* has immersed fruiting bodies, beneath light brown circular regions with a blackened outline, with 1–4 ostioles, *N. pennies* has comparatively smaller ascospores ((52) 57–64 (-84) × 2.5–3), *N. phayaoense* has hyaline to pale brown ascospores, and tapering towards the base (Hyde et al. 1998; Hyde and Alias 1999; Bhilabutra et al. 2006; Vitoria et al. 2013; Senwanna et al. 2018). Therefore, we identify our collection as a new species from Yunnan Province, China.

##### Neoleptosporellaceae

Taxon classificationFungiThyridialesNeoleptosporellaceae

J.F. Zhang, Y.Y. Chen & Jian K. Liu, Fungal Diversity 122: 110 (2023)

06947581-5007-56CA-8A65-37870853FD73

###### Notes.

Neoleptosporellaceae was introduced by Zhang et al. (2023a) to accommodate *Neoleptosporella* as the type genus. Members of Neoleptosporellaceae are saprobes and they are reported on various plant hosts in tropical and temperate regions (Phukhamsakda et al. 2020; Hyde et al. 2020a; Zhang et al. 2023a, 2024). The sexual morph is characterized by solitary and immersed, brown to black, coriaceous, and subglobose to depressed globose ascomata, the asci remain unitunicate and broadly cylindrical, possessing a pedicellate form along with a J-, wedge-shaped, subapical ring. The ascospores are fasciculate, fusiform, C-shaped, or sigmoid configurations, non-septate, with acute ends and a guttulate appearance (Zhang et al. 2023a). The asexual morph of the family has not been observed.

##### Neoleptosporella camporesiana

Taxon classificationFungiThyridialesNeoleptosporellaceae

R.H. Perera & K.D. Hyde, Fungal Diversity 100: 219 (2020)

988DDEAB-E08B-5D37-B655-FE2DC1B81529

Index Fungorum: IF556898

Facesoffungi Number: FoF06962

Fig. 9

###### Description.

***Saprobic*** on dead woody twigs. **Sexual morph**: Appearing as shiny black, raised dome-shaped spots, with a central short papilla. ***Ascomata*** 180–250 μm high, 195–300 μm diam. (x̄ = 220 × 250 μm, n = 5), solitary or aggregated, immersed beneath small clypeus appearing as a disc around the neck, uni-loculate, subglobose to depressed globose, ostiolate. ***Peridium*** 6–10 μm wide, outer cells fused with the host epidermal cells, composed of dark brown cells of ***textura angularis***. ***Paraphyses*** 3–7 µm diam., hyaline, branched, septate. ***Asci*** 75–100 × 7–9 μm (x̄ = 82 × 8 μm, n = 20), 8-spored, unitunicate, cylindrical, short pedicellate, apex rounded with a wedge-shaped, J- apical ring. ***Ascospores*** 60–74 × 2.1–2.6 μm (x̄ = 58 × 2.3 μm, n = 30), fasciculate, parallel becoming spiral at maturity, fliform, straight or curved, hyaline, aseptate, rounded at the apex, pointed at the base, smooth-walled, without append ages. **Asexual morph**: Undetermined.

**Figure 9.**
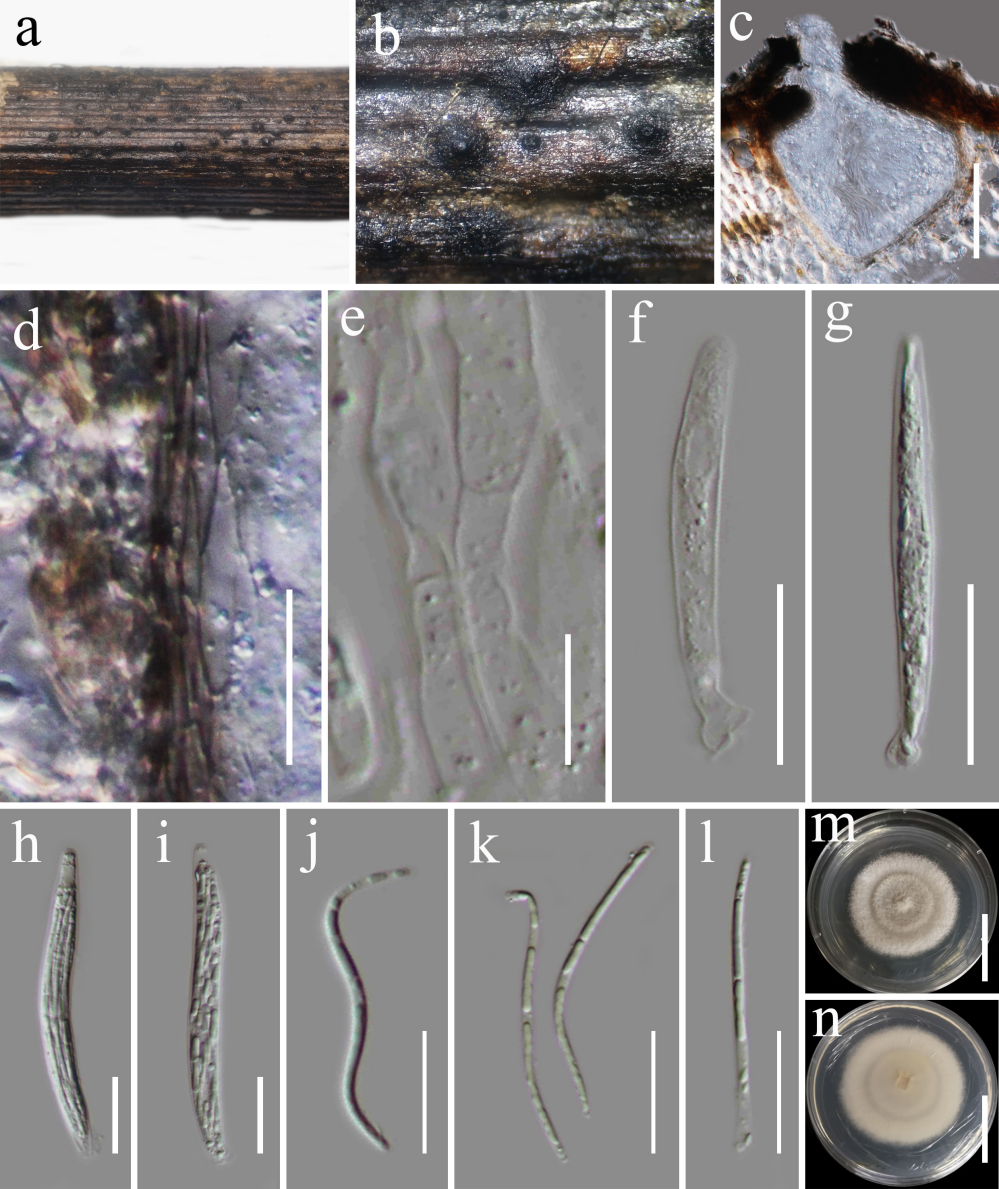
*Neoleptosporella camporesiana* (HKAS 122885). **a, b**. Appearance of ascomata on host substrate; **c**. Section of ascoma; **d**. Peridium; **e**. Pseudoparaphyses; **f–i**. Asci; **j–l**. Ascospores; **m, n**. Culture characters on PDA (**m** from above; **n** from below). Scale bars: 100 μm (**c**); 20 μm (**d**); 10 μm (**e**); 20 μm (**f–l**); 20 mm (**m, n**).

###### Culture characteristics.

Ascospores germinating on PDA within 24 h at room temperature (25 °C). Colonies on PDA, reaching 30–40 mm diameter after one week at 20–25 °C, mycelia superficial, circular, flat, fimbriate, entire edge, white; reverse, white.

###### Material examined.

China • Yunnan Province, Lincang (24°5'30"N, 100°5'33"E, elevation: 1557.49 m), on dead woody twigs of *Castanopsis mekongensis* (Fagaceae), 12 July 2020, G.C. Ren, LC40 (HKAS 122881), living culture KUNCC 21-0612; • *ibid*., Xianggelila, tacheng (27°25'30"N, 99°50'44"E, elevation: 3190 m), on dead woody twigs of *Sorbus rehderiana* (Rosaceae), 1 September 2020, G.C. Ren, T709 (HKAS 122885), living culture KUNCC 21-0655.

###### Known distribution and hosts.

Thailand (Hyde et al. 2020a; Phukhamsakda et al. 2020; Chaiwan et al. 2021), China (Manawasinghe et al. 2022; Chethana et al. 2023; this study). On dead stems of *Clematis subumbellata* (Phukhamsakda et al. 2020), dead branch of an unidentified plant (Hyde et al. 2020a; Chethana et al. 2023), dead stem of *Heteropanax fragrans* (Manawasinghe et al. 2022) and *Dracaena* sp. (Jayasiri et al. 2015), on dead woody twigs of *Castanopsis mekongensis* (Fagaceae) and *Sorbus rehderiana* (Rosaceae) (This study).

###### GenBank numbers.

KUNCC 21-0612: ITS: PX568861, LSU: PX473153, SSU: PX583843, *tef*1-α: PX521733. KUNCC 21-0655: ITS: PX568862, LSU: PX473154, *tef*1-α: PX521734.

###### Notes.

*Neoleptosporella* was proposed by Phukhamsakda et al. (2020) and typified with *N. clematidis*. Four species are accepted in *Neoleptosporella*, *Neoleptosporella camporesiana*, *N. clematidis*, *N. fusiformispora*, *N. palmae* (Hyde et al. 2020a; Phukhamsakda et al. 2020; Zhang et al. 2023a; Zhang et al. 2024). According to the multi-gene phylogenetic analyses of combined SSU, LSU, ITS, and *tef*1-α sequence dataset, our new isolates (KUNCC 21-0612, KUNCC 21-0655) nested with the strains of *Neoleptosporella camporesiana* (ZHKUCC 22-0218, MFLUCC 15-1016, Tak33) with 100% ML and 1.00 BYPP bootstrap support (Fig. 7). Our collection is morphologically similar to *Neoleptosporella camporesiana* in having ascomata with black shiny ostioles, cylindrical asci with J- apical ring and filiform ascospores (Hyde et al. 2020a). Sequence comparison for the ITS and *tef*1-α region between our isolates (KUNCC 21-0612, KUNCC 21-0655) and *Neoleptosporella camporesiana* (MFLUCC 15-1016) showed no significant base pair differences. Therefore, we identified our taxon as a new host record of *Neoleptosporella camporesiana* from *Castanopsis mekongensis* (Fagaceae) and *Sorbus rehderiana* (Rosaceae) in China.

#### Planisphaeriales J.F. Zhang, Jian K. Liu & K.D. Hyde, 2023

##### Planisphaeriaceae

Taxon classificationFungiPlanisphaerialesPlanisphaeriaceae

J.F. Zhang, Jian K. Liu & K.D. Hyde, Fungal Diversity 222: 114 (2023)

DA1F9C5A-AB2E-54AB-A03D-E0C4BDFCF742

###### Notes.

Planisphaeriaceae was established by Zhang et al. (2023b) to accommodate the genus *Planisphaeria*, with *Planisphaeria reniformispora* designated as the type species. The family was delineated based on morphological characteristics and phylogenetic analyses using LSU, SSU, *tef*1-α, and *rpb*2 sequence data from species within Sordariomycetes. Currently, Planisphaeriaceae comprises a single genus, *Planisphaeria*, which includes two species, *P. reniformispora* and *P. karsti*. Members of this family are saprophytic and occur on dead woody plants (Zhang et al. 2023b; Hyde et al. 2024b). In this study, we introduced two new host records, *Planisphaeria karsti* and *P. reniformispora* from *Trigonobalanus doichangensis* (Fagaceae).

##### Planisphaeria karsti

Taxon classificationFungiPlanisphaerialesPlanisphaeriaceae

J.F. Zhang & K.D. Hyde, Fungal Diversity 222: 117 (2023)

53E6A0BD-6619-5B55-8721-1A61306DE396

Index Fungorum: IF900742

Facesoffungi Number: FoF14598

Fig. 11

###### Description.

***Saprobic*** on dead woody twigs. **Sexual morph: *Ascomata*** 300–400 µm high, 220–250 µm diam. (x̄ = 345 × 240 µm, n = 5), immersed to erumpent, solitary or scattered, subglobose to globose, fattened at the base, glabrous, black, coriaceous. ***Ostiole*** central, 105–160 µm long, 50–75 µm diam. (x̄ = 138 × 63 µm, n = 5), immersed, long papillate, hyaline hyphae with periphyses. ***Peridium*** 10–14 μm wide, composed of dark brown, thick-walled cells of ***textura angularis*** that fused with host tissues. ***Hamathecium*** 2.5–4.5 μm wide, comprising abundant, branched, septate, hyphae like paraphyses, anastomosed. ***Asci*** 80–110 × 23–30 μm (x̄ = 94 × 27 μm, n = 20), 8-spored, unitunicate, broadly clavate, short pedicellate, rounded to truncate at the apex, with an inconspicuous apical ring. ***Ascospores*** 22–27 × 7–10 μm (x̄ = 24 × 8 μm, n = 30), overlapping, biseriate, hyaline, reniform to allantoid, aseptate, rounded at both ends, curved, guttulate, verrucose, without mucilaginous sheath or appendages. **Asexual morph**: Undetermined.

**Figure 10.**
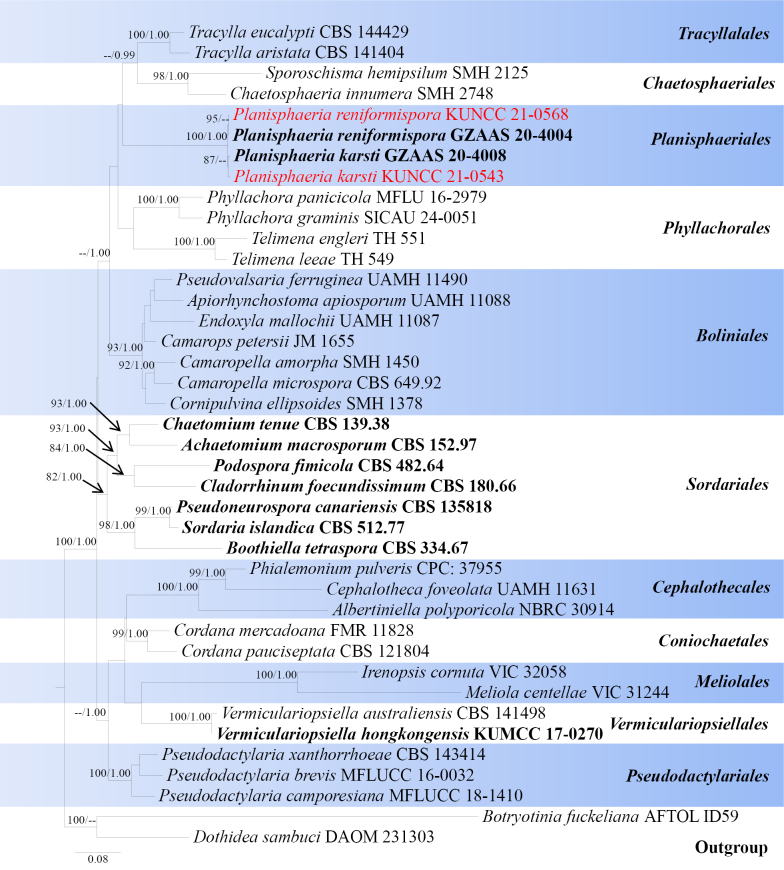
Phylogram generated from ML analysis based on LSU, SSU, ITS, *tef*1-α and *rpb*2 sequence data representing Sordariomycetes. Related sequences are obtained following Zhang et al. (2023b). Forty strains are included in the combined analyses, which comprise 4105 characters for LSU, SSU, ITS, *tef*1-α and *rpb*2 alignment. *Botryosphaeria fuckeliana* (AFTOLID59) and *Dothidea sambuci* (DAOM 231303) were used as the outgroup taxa. The best-scoring RAxML tree with a final likelihood value of -28303.773266 is presented. The matrix had 1766 distinct alignment patterns, with 54.76% of undetermined characters or gaps. Estimated base frequencies were as follows; A = 0.243984, C = 0.250047, G = 0.288655, T = 0.217314; substitution rates AC = 1.401636, AG = 2.371260, AT = 1.506416, CG = 1.236045, CT = 6.334641, GT = 1.0000. Gamma distribution shape parameter α = 0.355387 and tree-length = 4.905723. The Bayesian analysis calculated the average standard deviation of split frequencies at the end of 2,000,000 MCMC generations as 0.006199. The tree topology of the ML analysis is similar to the Bayesian analysis. Bootstrap values for ML equal to or greater than 70% and posterior probability values greater than 0.95 from BYPP analysis labelled on the nodes. Strains of the newly described species are in red, while type strains are in bold.

**Figure 11.**
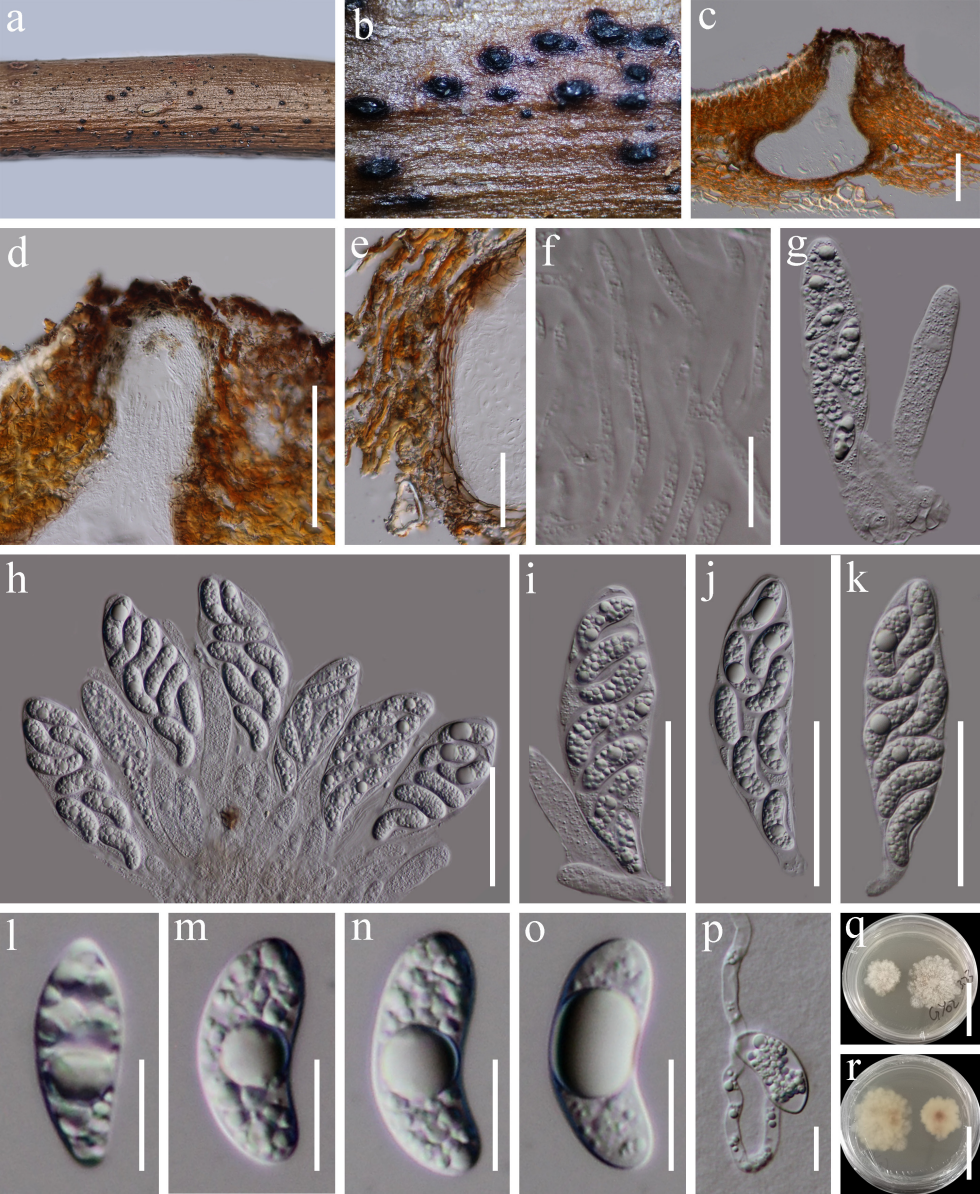
*Planisphaeria karsti* (HKAS 122721). **a, b**. Appearance of ascomata on host substrate; **c**. Section of ascoma; **d**. Section of ostiole; **e**. Peridium; **f**. Paraphyses; **g–k**. Asci; **l–o**. Ascospores; **p**. A germinating ascospore; **q, r**. Culture characters on PDA (**q** from above; **r** from below). Scale bars: 100 μm (**c, d**); 50 μm (**e**); 20 μm (**f**); 50 μm (**g–k**); 10 μm (**l–p**); 20 mm (**q, r**).

###### Culture characteristics.

Ascospores germinated on PDA within 24 h at room temperature (25 °C). Colonies on PDA, reaching 20 mm diameter after one week at 20–25 °C, mycelia superficial, irregular, flat, fimbriate, undulate, white; reverse, white, sometimes yellowish at the center.

###### Material examined.

China • Yunnan Province, Lancang, (24°5'30"N, 100°5'33"E, elevation: 1557.49 m), on dead woody twigs of *Trigonobalanus doichangensis* (Fagaceae), 19 July 2020, G.C. Ren, GY02 (HKAS 122721), living culture KUNCC 21-0543.

###### GenBank numbers.

ITS: PX568863, LSU: PX473155, SSU: PX583844, *tef*1-α: PX521735, *rpb*2: PX521739.

###### Known hosts and distribution.

China, on dead branch of woody plant (Zhang et al. 2023b), on dead woody twigs of *Trigonobalanus doichangensis* (Fagaceae) (this study).

###### Notes.

According to the multi-gene phylogenetic analyses of combined LSU, SSU, ITS, *tef*1-α, and *rpb*2 sequence dataset, our new isolate (KUNCC 21-0543) nested with *Planisphaeria karsti* (GZAAS 20-4008), which was isolated from dead branch of woody plant in China (Zhang et al. 2023b) with 87% ML bootstrap support (Fig. 10). The new collection shares similar morphology with the type material of *Planisphaeria karsti* in having subglobose to globose, fattened as the base, black ascostromata, periphyses ostiole, broadly clavate asci with short pedicellate and an inconspicuous apical ring, hyaline, one-celled, aseptate, guttulate, verrucose ascospores. However, there is a difference in the shape of the spores, *Planisphaeria karsti* has irregular ellipsoid ascospores tapered towards one end and rounded at another end, whereas the new collection has reniform to allantoid ascospores with rounded ends. Sequence comparison for the LSU and SSU region between our isolates (KUNCC 21-0543) and *P. karsti* (GZAAS 20-4008) showed no significant base pair differences, 1.1% (11/ 1037 bp, without gaps) base pair difference for *rpb*2 region, but we were unable to compare ITS and *tef*1-α gene of *P. karsti* as there was no sequence data. We report our collection as a new host record of *P. karsti* from *Trigonobalanus doichangensis* (Fagaceae) in China.

##### Planisphaeria reniformispora

Taxon classificationFungiPlanisphaerialesPlanisphaeriaceae

J.F. Zhang & K.D. Hyde, Fungal Diversity 222: 117 (2023)

4D0B5AFA-300C-5545-A398-D7D0D5291058

Index Fungorum: IF900741

Facesoffungi Number: FoF14597

Fig. 12

###### Description.

***Saprobic*** on dead woody twigs. **Sexual morph: *Ascomata*** 300–380 μm high, 200–370 μm diam. (x̄ = 340 × 280 μm, n = 5), immersed to erumpent, solitary or scattered, uni-loculate, subglobose to obpyriform, fattened at the base, black, coriaceous, central ostiole. ***Peridium*** 13–20 μm wide, composed of brown, thick-walled cells of ***textura angularis*** that merge with host tissue. ***Hamathecium*** 3–6 μm wide, comprising abundant, branched, septate, hyphae-like paraphyses, anastomosed. ***Asci*** 60–87 × 16–24 μm (x̄ = 72 × 20 μm, n = 20), 8-spored, unitunicate, clavate to broadly clavate, with a short pedicel, rounded to truncate at the apex, with an inconspicuous apical ring. ***Ascospores*** 19–23 × 8–10 μm (x̄ = 21 × 9 μm, n = 30), overlapping biseriate, hyaline, mostly irregular ovoid, straight, tapered towards one end and rounded at other end, aseptate, thick-walled, guttulate, verrucose, without mucilaginous sheath or appendages. **Asexual morph**: Undetermined.

**Figure 12.**
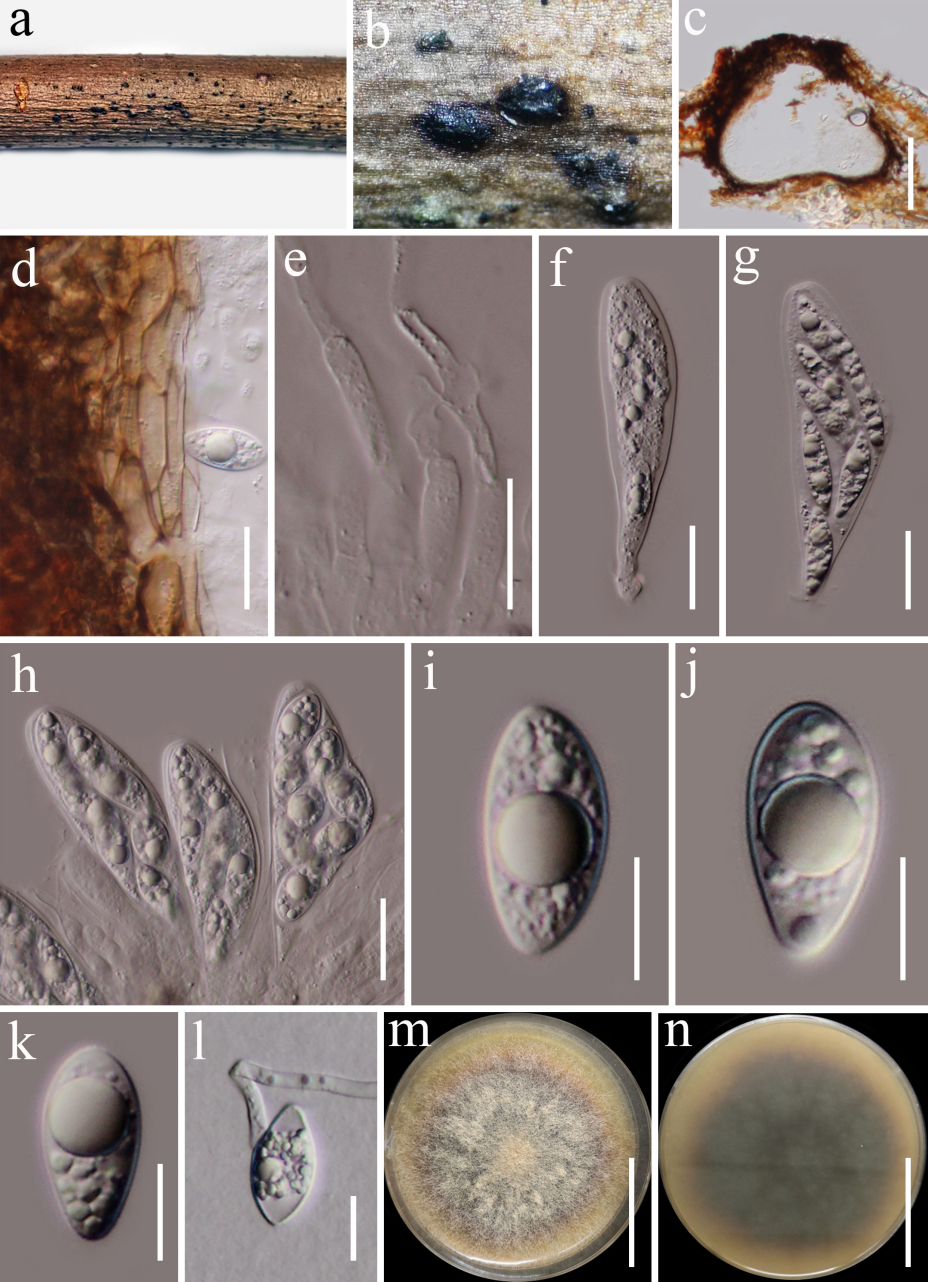
*Planisphaeria reniformispora* (HKAS 122722). **a, b**. Appearance of ascomata on host substrate; **c**. Section of ascoma; **d**. Peridium; **e**. Paraphyses; **f–h**. Asci; **i–k**. Ascospores; **l**. A germinating ascospore; **m, n**. Culture characters on PDA (**m** from above; **n** from below). Scale bars: 150 μm (**c**), 20 μm (**d–h**), 10 μm (**i–l**), 20 mm (**m, n**).

###### Culture characteristics.

Ascospores germinated on PDA within 24 h at room temperature (25 °C). Colonies on PDA, reaching 50 mm diameter after two weeks at 20–25 °C, mycelia superficial, circular, sparse mycelia, flat, fimbriate, white; reverse, dark brown.

###### Material examined.

China • Yunnan Province, Lancang, (24°5'30"N, 100°5'33"E, elevation: 1557.49 m), on dead woody twigs of *Trigonobalanus doichangensis* (Fagaceae), 19 July 2020, G.C. Ren, GY42 (HKAS 122722), living culture KUNCC 21-0568.

###### GenBank numbers.

ITS: PX568864, LSU: PX473156, SSU: PX583845, *tef*1-α: PX521736.

###### Known hosts and distribution.

China, on dead branch of unidentified woody plant (Zhang et al. 2023b); on dead woody twigs of *Trigonobalanus doichangensis* (Fagaceae) (this study).

###### Notes.

According to the multi-gene phylogenetic analyses of combined LSU, SSU, ITS, *tef*1-α, and *rpb*2 sequence dataset, our new isolate (KUNCC 21-0568) nested with *Planisphaeria reniformispora* (GZAAS 20-4004), which was isolated from dead branch of woody plant in China (Zhang et al. 2023b) with 95% ML bootstrap support (Fig. 10). The new collection shares similar morphology with the type material of *Planisphaeria reniformispora* in having immersed to erumpent, subglobose to obpyriform, fattened at the base, black ascomata, clavate asci and hyaline, one-celled, guttulate, verrucose ascospores. However, there is a difference in the shape of the spores, *Planisphaeria reniformispora* in having reniform ascospores, whereas the new collection has irregular ovoid ascospores. Sequence comparison for the LSU and SSU region between our isolates (KUNCC 21-0568) and *P. reniformispora* (GZAAS 20-4004) showed no significant base pair differences. Therefore, we identified our taxon as a new host record of *P. reniformispora* from *Trigonobalanus doichangensis* (Fagaceae) in China.

#### Sordariales Chadef. ex D. Hawksw. & O.E. Erikss., 1986

##### Lasiosphaeridaceae

Taxon classificationFungiSordarialesLasiosphaeridaceae

S.K. Huang, Maharachch. & K.D. Hyde, Fungal Diversity, 111: 443–572 (2021).

1842C8CC-D4AC-513F-9B9B-C3ADCD07DEB4

###### Notes.

Lasiosphaeridaceae was established by Huang et al. (2021) in Sordariales to accommodate the monotypic genus *Lasiosphaeris*, based on both morphological characteristics and multigene phylogenetic analyses of LSU, ITS, TUB and *rpb*2 sequence data. Currently, the genus comprises four species, *L. arenicola* (=*Cercophora arenicola*), *L. hispida* and *L. hirsuta* and *L. indica* (Huang et al. 2021). In this study we introduced a new host record of *Lasiosphaeris hispida*.

##### Lasiosphaeris hispida

Taxon classificationFungiSordarialesLasiosphaeridaceae

(Tode) Clem., Gen. fung. (Minneapolis): [173] (1909)

729965DD-F655-5FD3-BE5F-B9CD7F1E45A1

Index Fungorum: IF531338

Facesoffungi Number: FoF10021

Fig. 14

###### Basionym.

*Sphaeria hispida* Tode, Fung. mecklenb. sel. (Lüneburg) 2: 17 (1791)

**Figure 13.**
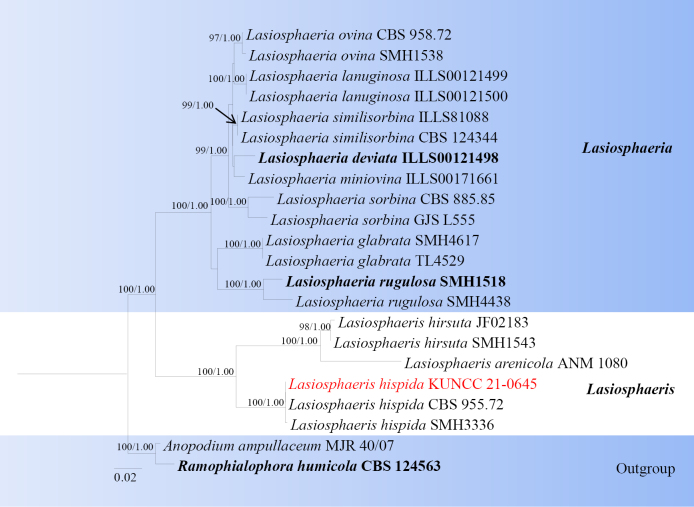
Phylogram generated from ML analysis based on LSU, ITS, and TUB sequence data representing *Lasiosphaeria* and *Lasiosphaeris*. Related sequences are obtained following Huang et al. (2021). Twenty-two strains are included in the combined analyses, which comprise 2669 characters for LSU, ITS and TUB alignment. *Anopodium ampullaceum* (MJR 40/07) and *Ramophialophora humicola* (CBS 124563) were used as the outgroup taxa. The best-scoring RAxML tree with a final likelihood value of -8642.701866 is presented. The matrix had 545 distinct alignment patterns, with 26.27% of undetermined characters or gaps. Estimated base frequencies were as follows; A = 0.230199, C = 0.259303, G = 0.278833, T = 0.231665; substitution rates AC = 1.180009, AG = 2.589188, AT = 1.230892, CG = 1.493571, CT = 9.951940, GT = 1.0000. Gamma distribution shape parameter α = 0.134824 and tree-length = 0.536057. The tree topology of the ML analysis is similar to the Bayesian analysis. Bootstrap values for ML equal to or greater than 70% and posterior probability values greater than 0.95 from BYPP analysis labelled on the nodes. Strains of the newly described species are in red, while type strains are in bold.

**Figure 14.**
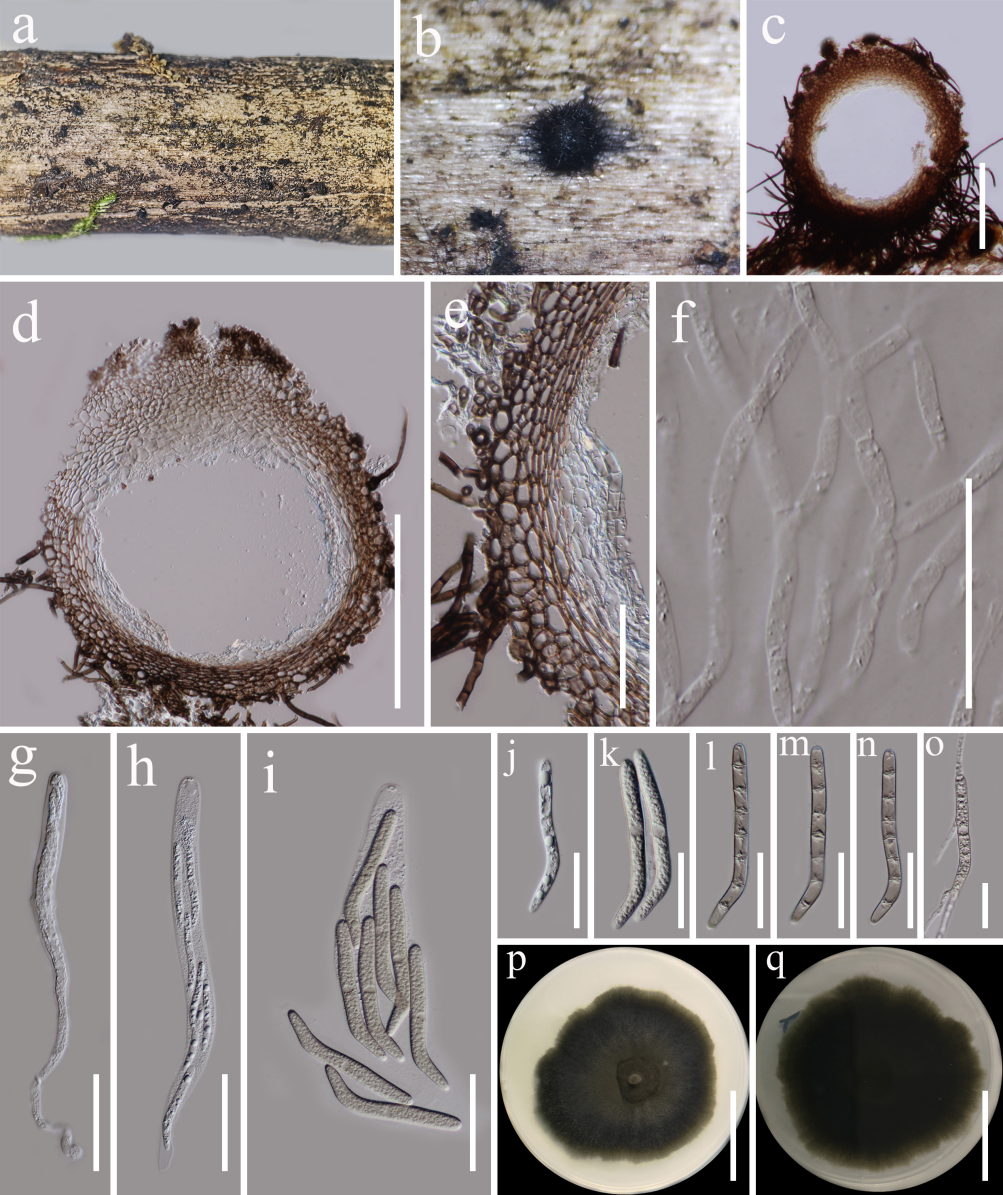
*Lasiosphaeris hispida* (HKAS 122766). **a, b**. Appearance of ascomata on host surface; **c, d**. Ascoma in cross section; **e**. Peridium; **f**. Paraphyses; **g–i**. Asci; **j–n**. Ascospores; **o**. Germinating ascospore; **p, q**. Culture characters on PDA (**p** from above; **q** from below). Scale bars: 200 µm (**c, d**); 50 µm (**e, f–i**); 30 µm (**j–o**); 20 mm (**p, q**).

###### Description.

***Saprobic*** on dead woody twigs. **Sexual morph: *Ascomata*** 380–460 μm high, 350–460 μm diam. (x̄ = 420 × 400 μm, n = 5), perithecial, solitary, superficial to semi-immersed, globose to subglobose, black, ostiolate, coriaceous, with numerous, septate, brown, setae (4–5 diam.). ***Peridium*** 40–56 µm (x̄= 50 µm, n = 30) wide, comprising two layers, outer layer composed of brown cells of ***textura angularis*** to ***textura prismatica***, inner layer composed of hyaline cells of ***textura angularis***. ***Paraphyses*** 3–5 µm wide, numerous, hyaline, filiform, septate, branched, slightly constricted at the septa, embedded in a gelatinous matrix. ***Asci*** 210–270 × 12–18 μm (x̄ = 250 × 15 μm, n = 20), 8-spored, unitunicate, cylindrical to clavate, long pedicellate, apex rounded, with apical globule, apical ring distinct. ***Ascospores*** 70–76 × 5.5–8 μm (x̄ = 73 × 6.7 μm, n = 30), bi-seriate, cylindrical, slightly curved near the base, hyaline and aseptate when young, becoming pale brown and 7-septate when mature, rounded at the apex, pointed at the base, guttule, smooth-walled. **Asexual morph**: Undetermined.

###### Culture characteristics.

Ascospores germinating on PDA within 24 h at room temperature (25 °C). Colonies on PDA, reaching 30–40 mm diameter after one week at 20–25 °C, mycelia superficial, irregular, flat, fimbriate, undulate entire, grey to atrovirens; reverse, atrovirens.

###### Material examined.

China • Yunnan Province, Nixi Diqing, Xianggelila, Nixi, (27°25'37"N, 99°50'45"E, elevation: 3090 m), on dead woody twigs of *Rhododendron rubiginosum* (Ericaceae), 1 September 2020, G.C. Ren, NX24 (HKAS 122766), living culture KUNCC 21-0645.

###### GenBank numbers.

ITS: PX568865, LSU: PX473157, SSU: PX583846.

###### Known hosts and distribution.

On dead wood in Germany (type locality) and USA (Fuckel 1870; Miller and Huhndorf 2005), on dead woody twigs of *Rhododendron rubiginosum* (Ericaceae) in China (this study).

###### Notes.

Multi-loci phylogenetic analyses based on a concatenated LSU, ITS, and TUB sequence dataset show that our new collections (KUNCC 21-0645) clusters with *Lasiosphaeris hispida* (CBS95572, SMH3336) with 100% ML and 1.00 BYPP bootstrap support (Fig. 13). Sequence comparison for the ITS, LSU and TUB region between our isolates (KUNCC 21-0645) and *Lasiosphaeris hispida* (SMH3336) showed no significant base pair differences. Our collection (KUNCC 21-0645) resembles *Lasiosphaeris hispida* in having sporodochial conidiomata; septate, subhyaline to light brown mycelium; mononematous, cylindrical, light brown conidiophores; monoblastic, terminal, light brown conidiogenous cells and muriform, oval to long elliptical conidia often with a hyaline, elliptical to globose, 0–multiple-basal cell (Huang et al. 2021). Therefore, we introduce our collection as the first record of *L. hispida* from *Rhododendron rubiginosum* (Ericaceae), and first time reported in China.

#### Xylariales Nannf., Nova Acta R. Soc. Scient. upsal., Ser. 4 8(no. 2): 66 (1932)

##### Barrmaeliaceae

Taxon classificationFungiXylarialesBarrmaeliaceae

Voglmayr & Jaklitsch, Mycol. Progr. 17(1–2): 162 (2018)

CBEC20E5-468D-57D4-A5D0-8041D0A5AA77

###### Notes.

Barrmaeliaceae is recently established by Voglmayr et al. (2018) based on multigene phylogenetic analyses using ITS, LSU, *rpb*2, and TUB sequences. The family was created to accommodate the type genus *Barrmaelia* and *Entosordaria*. Hyde et al. (2024b) proposed that *Induratia*, previously placed in the family Induratiaceae (Samarakoon et al. 2020), should be accommodated in Barrmaeliaceae due to its closer phylogenetic affinity with this family. In this study we introduced a new species *Barrmaelia nixiensis*.

##### Barrmaelia nixiensis

Taxon classificationFungiXylarialesBarrmaeliaceae

G.C Ren & Wanas. sp. nov.

4EAA97DA-390C-5440-AEDD-D0175FEE3B58

Index Fungorum: IF904526

Facesoffungi Number: FoF18854

Fig. 16

###### Etymology.

The specific epithet “nixiensis” reflects nixi where the holotype was collected.

**Figure 15.**
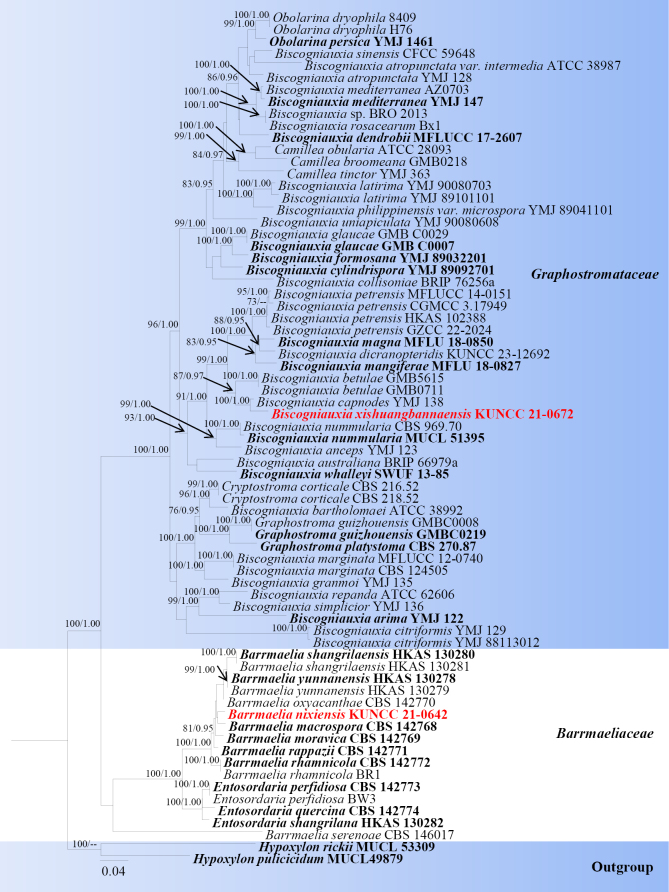
Phylogram generated from ML analysis based on LSU, ITS, *tef*1-α, *rpb*2 and TUB sequence data representing Barrmaeliaceae and Graphostromataceae. Related sequences are obtained following Chen et al. (2024) and GenBank (http://www.ncbi.nlm.nih.gov). Seventy-one strains are included in the combined analyses, which comprise 5409 characters for LSU, ITS, *tef*1-α, *rpb*2 and TUB alignment. *Hypoxylon pulicicidum* (MUCL49879) and *H. rickii* (MUCL 53309). were used as the outgroup taxa. The best-scoring RAxML tree with a final likelihood value of -44661.622236 is presented. The matrix had 2258 distinct alignment patterns, with 45.28% of undetermined characters or gaps. Estimated base frequencies were as follows; A = 0.236549, C = 0.271101, G = 0.252706, T = 0.239644; substitution rates AC = 1.148601, AG = 4.023809, AT = 1.100668, CG = 0.916979, CT = 6.158610, GT = 1.0000. Gamma distribution shape parameter α = 0.244516 and tree-length = 3.234831. The tree topology of the ML analysis is similar to the Bayesian analysis. Bootstrap values for ML equal to or greater than 70% and posterior probability values greater than 0.95 from BYPP analysis labelled on the nodes. Strains of the newly described species are in red, while type strains are in bold.

**Figure 16.**
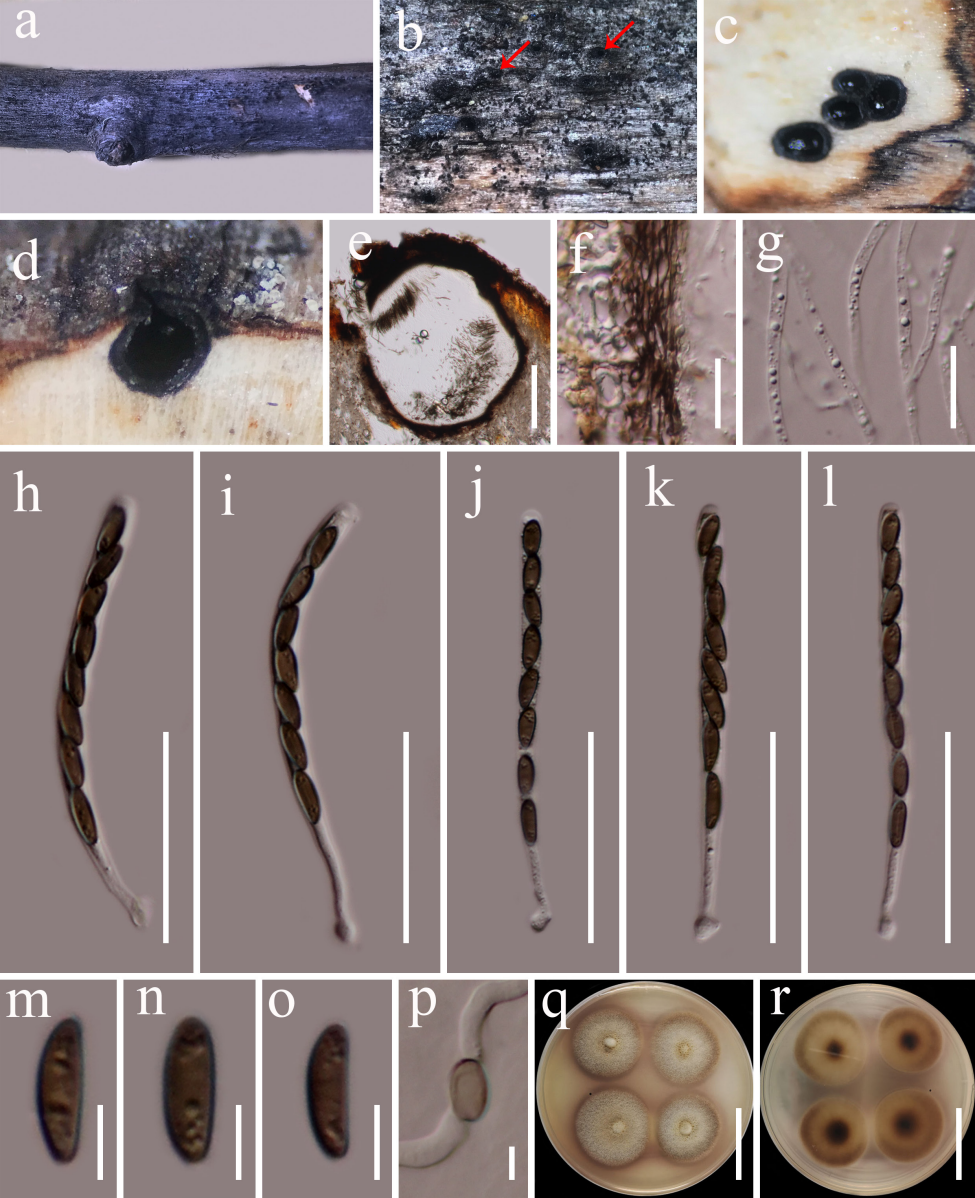
*Barrmaelia nixiensis* (HKAS 122884, holotype). **a, b**. Appearance of ascomata on the host substrate (red arrows indicate ascomata); **c**. Ascomata in transverse section; **d**. Ascoma in vertical section; **e**. Section of the ascoma; **f**. Peridium; **g**. Paraphyses; **h**–**l**. Asci; **m**–**o**. Ascospores; **p**. A germinated ascospore; **q, r**. Culture characters on PDA (**q** from above; **r** from below). Scale bars: 200 μm (**e**); 20 μm (**f, g**); 50 μm (**h–l**); 5 μm (**m–p**); 30 mm (**q, r**).

###### Holotype.

HKAS 122884

###### Description.

***Saprobic*** on dead woody twigs. **Sexual morph: *Ascomata*** 450–600 μm high, 450–530 μm diam. (x̄ = 520 × 490 μm, n = 5), immersed in bark, solitary or aggregated, globose to subglobose, uni-loculate, coriaceous, visible as black dots, ostiolar openings on the stromatal surface. ***Ostioles*** centric, often elongate, ostiolar pore rounded, shiny and slightly raised. ***Peridium*** 12–18 μm thick, composed of brown cells of ***textura angularis***, thick–walled. ***Paraphyses*** 2.5–4 μm wide, abundant, hyaline, filamentous, sinuous, unbranched, septate, guttulate, slightly tapering towards the apex, obtuse. ***Asci*** 75–95 × 5–6 μm (x̄ = 90 × 5.7 μm, n = 20), 8-spored, unitunicate, cylindrical, straight or curved, long pedicellate, apically rounded. ***Ascospores*** 8.5–11 × 3.2–4 μm (x̄ = 9.8 × 3.7 μm, n = 30), uniseriate, unicellular, ellipsoidal to oblong, straight, brown, small guttules, smooth-walled, without a gelatinous sheath and without germ slit. **Asexual morph**: Undetermined.

###### Culture characteristics.

Ascospores germinating on PDA within 24 h at room temperature (25 °C). Germ tubes are produced from the basal and apical cell of ascospore. Colonies on PDA, reaching 20 mm diameter after one week at 20–25 °C, mycelia superficial, raised, circular, fimbriate, dense, flat, entire edge, white to light brown; reverse, light brown at the edge, dark brown at the center.

###### Material examined.

China • Yunnan Province, Diqing, Xianggelila, Nixi, (27°25'37"N, 99°50'45"E, elevation: 3090 m), on dead woody twigs of *Rhododendron rubiginosum* (Ericaceae), 31 August 2020, G.C. Ren, NX17 (holotype, HKAS 122884), ex-type culture KUNCC 21-0642.

###### GenBank numbers.

ITS: PP663076, LSU: PP663082, *tef*1-α: PX521737.

###### Notes.

Multi-loci phylogenetic analyses based on a concatenated LSU, ITS, *tef*1-a and *rpb*2 dataset show that our new collections (KUNCC 21-0642) clusters with *Barrmaelia macrospora* (CBS 142768), but they did not receive statistical support values (Fig. 15). Sequence comparison for the ITS region between *Barrmaelia nixiensis* (KUNCC 21-0642) and *Barrmaelia macrospora* (CBS 142768) showed a 1.86% (10/ 538 bp, without gaps) base pair difference, 2% (15/ 751 bp, without gaps) base pair difference for *tef*1-a region. Morphologically, *Barrmaelia nixiensis* differs from *B. macrospora* in having comparatively smaller asci (75–95 × 5–6 vs. 108–143 × 9–11) with long pedicellate, smaller asci ascospores (8.5–11 × 3.2–4 vs. (18.2–)20.5–24.0(−26.0) × (4.0–)4.8–5.9(−6.5)), while *B. macrospora* has larger asci with short stipe and larger ascospores with a lighter coloured band at the concave side and germ slit (Voglmayr et al. 2018). Based on morphology and the phylogenetic distinctiveness, we introduce *Barrmaelia nixiensis* as a new species.

##### Graphostromataceae

Taxon classificationFungiXylarialesGraphostromataceae

M.E. Barr, J.D. Rogers & Y.M. Ju, Mycotaxon 48: 533 (1993)

5EF6D3B4-E36B-5417-A28D-367CA759B524

###### Notes.

Graphostromataceae was introduced by Barr et al. (1993), which includes saprobes, endophytes and pathogens on numerous plant species globally (Chen et al. 2024). Currently, five genera are recognized within the family, Biscogniauxia, Camillea, Graphostroma, Obolarina and Vivantia (Hyde et al. 2024b). In this study, we introduced a new species, *Biscogniauxia xishuangbannaensis*.

##### Biscogniauxia xishuangbannaensis

Taxon classificationFungiXylarialesGraphostromataceae

G.C Ren & Wanas. sp. nov.

ECFCC074-B546-5092-87CB-AC444AF80366

Index Fungorum: IF904527

Facesoffungi Number: FoF18855

Fig. 17

###### Etymology.

The specific epithet “xishuangbannaensis” reflects xishuangbanna where the holotype was collected.

**Figure 17.**
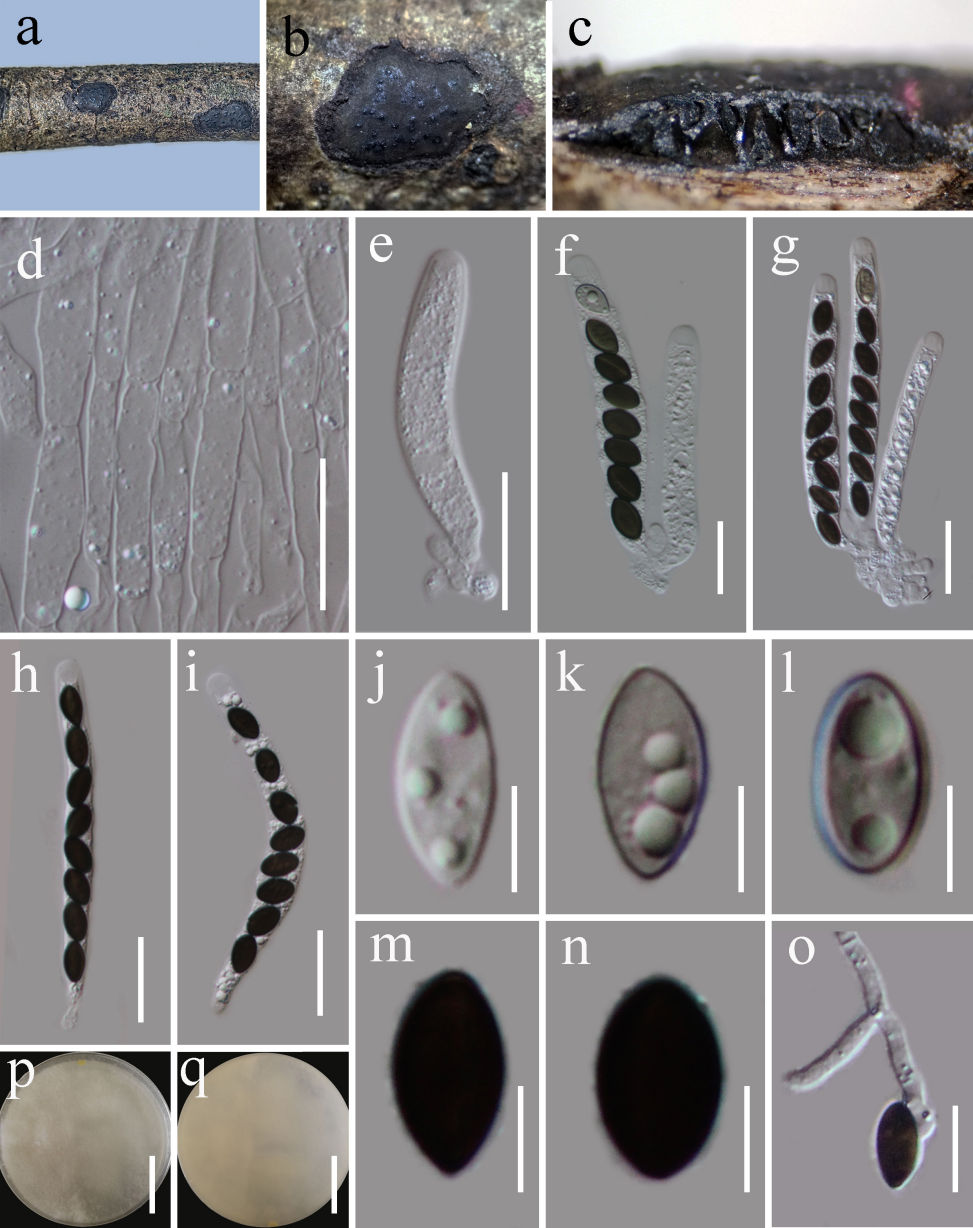
*Biscogniauxia xishuangbannaensis* (HKAS 122774, holotype). **a, b**. Appearance of stromata on the host substrate; **c**. Ascomata in vertical section; **d**. Paraphyses; **e–i**. Asci; **j–n**. Ascospores; **o**. A germinated ascospore; **p, q**. Culture characters on PDA (**p** from above; **q** from below). Scale bars: 20 μm (**d–i**); 5 μm (**j–n**); 10 μm (**o**), 20 mm (**p, q**).

###### Holotype.

HKAS 122774.

###### Description.

***Saprobic*** on dead woody twigs. **Sexual morph: *Stromata*** 3–18 × 2–7 mm, superficial, aggregated, applanate, cover the wood surface, dull brown to black, carbonaceous, visible as black dots on the stromata. ***Ascomata*** 300–480 × 250–400 μm (x̄ = 360 × 315 μm, n = 5), ovoid to obpyriform, laterally compressed. ***Ostioles*** pointed, raised. ***Paraphyses*** 3–7 μm wide, long, numerous, filamentous, flexuous, septate, constricted at the septa, apex rounded, slightly tapering towards the apex. ***Asci*** 75–95 × 5.7–9 μm (x̄ = 86 × 7.7 μm, n = 20), 8-spored, unitunicate, cylindrical, short pedicellate, with a 5–6.6 × 3.5–5 μm (x̄ = 5.8 × 4.2 μm, n = 20), wedge-shaped apical ring, apex rounded. ***Ascospores*** 8–11 × 4.4–6 μm (x̄ = 10 × 5 μm, n = 30), uniseriate, unicellular, ellipsoid, rounded ends, hyaline when immature, dark brown to black when mature, 1–2-guttulate, aseptate, with straight germ slit along the entire spore length. **Asexual morph**: Undetermined.

###### Culture characteristics.

Ascospores germinating on PDA within 24 hrs at room temperature. Germ tubes are produced from the basal cells of the ascospore. Colonies on PDA, reaching 55 mm diameter after one week at 20–25 °C, mycelia superficial, circular, dense, fimbriate, entire edged, white; reverse view white.

###### Material examined.

China • Yunnan Province, Xishuangbanna, Xishuangbanna Dai Autonomous Prefecture, Jinghong (21°55.19'N, 101°15.24'E, elevation: 522 m), on dead woody twigs of undetermined tree, 15 July 2019, G.C Ren, XS19 (holotype, HKAS 122774), ex-type culture KUNCC 21-0672.

###### GenBank numbers.

ITS: PP663077, LSU: PP663083, SSU: PX583847, *tef*1-α: PX521738, *rpb*2: PX521740.

###### Notes.

Multi-loci phylogenetic analyses based on a concatenated LSU, ITS, *tef*1-a, and *rpb*2 dataset show that our new strain (KUNCC 21-0672) clustered with *Biscogniauxia capnodes* (YMJ 138) with 100% ML and 1.00 BYPP bootstrap support (Fig. 15). Sequence comparison for the ITS region between *Biscogniauxia xishuangbannaensis* (KUNCC 21-0672) and *Biscogniauxia capnodes* (YMJ 138) showed a 2.49% (15/ 602 bp, without gaps) base pair difference, 3.15% (32/ 1015 bp, without gaps) base pair difference for *rpb*2 region. *Biscogniauxia capnodes* differs from our strain by its elongated, erumpent patches, obovoid to tubular perithecia, discoid apical ring and large ascospores (Berkeley 1845; Ju and Rogers 2001). Based on morphology and the phylogenetic distinctiveness, we introduce *Biscogniauxia xishuangbannaensis* as a new species.

## Discussion

Over the past few decades, research on fungal diversity in China has expanded substantially (Dai et al. 2015; Wang and Cai 2023). This progress reflects the broad climatic gradients and heterogeneous ecosystems in China, which provide diverse habitats that support high fungal richness (Ding et al. 2025). Wang and Cai (2023) reported that 15,626 fungal taxa have been newly described from China, and that species recorded from China for the first time represent 6.84% of the currently documented global fungal diversity, placing China among the leading contributors worldwide. Newly described taxa are disproportionately reported from southwestern China (particularly Guizhou, Sichuan, Tibet and Yunnan) where complex topography and strong environmental gradients promote diversification and endemism (Yu et al. 2022; Zhang et al. 2023b; Hongsanan et al. 2023; Sun et al. 2024; Chen et al. 2025; Phurbu et al. 2025; Shen et al. 2025; Dissanayake et al. 2024a; Wanasinghe and Maharachchikumbura 2023; Wanasinghe et al. 2025). In addition, low-latitude tropical and subtropical regions, including Taiwan and Guangdong, also contribute substantially to national fungal diversity and continue to yield numerous novel and newly recorded taxa (Cheng et al. 2025; de Silva et al. 2025). In the present study, we focused on saprobic Sordariomycetes from woody litter in Yunnan, and based on morphological characteristics and multi-gene phylogenetic analyses, we introduce three new species and seven new records.

This study represents the first report of *Jattaea algeriensis* from China, where it was collected on dead woody twigs of *Myrsine seguinii* (Myrsinaceae). This species has previously been recorded in Algeria on decayed canes of *Rubus fruticosus*, South Africa on wood of *Prunus salicina* (Saccardo and Sydow 1902; Damm et al. 2008; Réblová 2011) and in Iran as a plant pathogen associated with *Mespilus germanica* and *Parrotia persica* (Kazemzadeh-Chakusary et al. 2020). The occurrence of *J. algeriensis* across geographically distant regions, contain North Africa, Southern Africa, the Middle East, and now East Asia, indicates that its distribution is likely broader than currently recorded and may be approaching a global scale. Given its confirmed pathogenicity on certain woody hosts in Iran, further understanding of the host range, environmental preferences, and transmission potential of *Jattaea algeriensis* is important for the prevention and control of the pathogen.

*Phaeoacremonium camporesii* (KUNCC 21-0608) and the ex-type strain of *P. camporesii* (MFLUCC 21-0224) exhibit a high degree of morphological similarity, particularly in the characteristics of the ascomata, asci, and ascospores. However, notable differences were observed in their stromatic development. The ex-type strain, *P. camporesii* (MFLUCC 21-0224), is capable of damaging the bark and forming conspicuous stromata on its surface, with numerous ascomata embedded within a single stroma. In contrast, our isolate (KUNCC 21-0608) does not produce stromata on the bark surface, and its ascomata occur independently. Molecular comparisons further support the close relationship between the two isolates, yet also reveal subtle genetic variation (Phukhamsakda et al. 2022; this study). Comparative sequence analysis of the ITS and TUB regions showed that the ITS region is identical between the two strains, indicating strong genetic affinity. However, the TUB region exhibited a 5.4% nucleotide difference. As β-tubulin is a protein-coding gene that often shows higher levels of variation than ribosomal markers, this divergence may reflect intraspecific variability rather than species-level separation. The morphological similarity, the absence of ITS divergence, and the observed differences in TUB suggest that KUNCC 21-0608 should be regarded as a conspecific isolate of *P. camporesii*. The observed variation in stromatic development may be attributable to differences in environmental conditions, host interaction, or intraspecific phenotypic plasticity. This finding also highlights the importance of integrating morphological, ecological, and multilocus phylogenetic data for accurate fungal identification and classification.

The family Planisphaeriaceae currently comprises a single genus, *Planisphaeria*, which includes two species, *P. reniformispora* and *P. karsti* (Hyde et al. 2024b). In the present study, we report *P. karsti* and *P. reniformispora* from *Trigonobalanus doichangensis* (Fagaceae) for the first time, thereby expanding their known host range. Morphological and phylogenetic analyses confirm that our isolate of *P. karsti* (KUNCC 21-0543) is conspecific with the previously described strain GZAAS 20-4008. Both share the key morphological features of the species, including ascomata structure, asci morphology, and ascospore characteristics, and they cluster together in multi-gene phylogenetic analyses. Nonetheless, the two strains differ in ascospore shape, and the size ranges of both asci and ascospores (Zhang et al. 2023b; this study). Notably, KUNCC 21-0543 possesses reniform to allantoid ascospores, whereas GZAAS 20-4008 produces irregular ellipsoid ascospores with asymmetrical ends. A similar pattern of morphological divergence is evident between *P. reniformispora* isolates. Our isolate (KUNCC 21-0543) resembles with the type and they are phylogenetically closely affiliated. However, key differences were detected as GZAAS 20-4004 has reniform ascospores surrounded by a distinct mucilaginous sheath, while our isolate bears irregular ovoid, verrucose ascospores without a sheath and with abundant paraphyses (Zhang et al. 2023b; this study). These observations highlight that while molecular phylogeny provides robust species delimitation within *Planisphaeria*, intraspecific morphological variability can be substantial. Such variability may be influenced by ecological, host-related, or environmental factors, and further sampling across different geographic regions and hosts is needed to assess the stability and taxonomic significance of these micromorphological differences.

*Barrmaelia* was established by Rappaz (1995) to accommodate *B. rhamnicola* as the type species. Currently, nine epithets are recorded in Index Fungorum (2026), with a global distribution across Africa, Asia, Europe, and North America (Voglmayr et al. 2018; Dissanayake et al. 2024b). Except for *Barrmaelia serenoae*, which occurs on leaves, all other known species inhabit dead branches and stalk (Voglmayr et al. 2018; Crous et al. 2020; Dissanayake et al. 2024b). The genus is known only from its sexual morph, however, Crous et al. (2020) reported an asexual morph for *B. serenoae* (as a new species). In their study, the fungus from *Serenoa repens* was tentatively assigned to *Barrmaelia*. In our phylogenetic analyses, *B. serenoae* occupies a basal position, distinct from the main *Barrmaelia* clade. This separation suggests that its phylogenetic placement within the genus remains uncertain and warrants further investigation. Moreover, the asexual morph of *Barrmaelia* is still poorly understood, further studies with additional collections are needed to understand the asexual morph.

## Supplementary Material

XML Treatment for Calosphaeriaceae

XML Treatment for Jattaea algeriensis

XML Treatment for Togniniaceae

XML Treatment for Phaeoacremonium camporesii

XML Treatment for Thyridiaceae

XML Treatment for Thyridium tiliae

XML Treatment for Linocarpaceae

XML Treatment for Neolinocarpon lincangense

XML Treatment for Neoleptosporellaceae

XML Treatment for Neoleptosporella camporesiana

XML Treatment for Planisphaeriaceae

XML Treatment for Planisphaeria karsti

XML Treatment for Planisphaeria reniformispora

XML Treatment for Lasiosphaeridaceae

XML Treatment for Lasiosphaeris hispida

XML Treatment for Barrmaeliaceae

XML Treatment for Barrmaelia nixiensis

XML Treatment for Graphostromataceae

XML Treatment for Biscogniauxia xishuangbannaensis

## Additional information

### Use of AI

No use of AI was reported.

### Author ORCIDs

Guang-Cong Ren https://orcid.org/0000-0001-9923-2626

Dhanushka Wanasinghe https://orcid.org/0000-0003-1759-3933

Heng Gui https://orcid.org/0000-0002-0946-1589

Turki Kh. Faraj https://orcid.org/0000-0002-6012-8474

### Data availability

All of the data that support the findings of this study are available in the main text.
